# Identification, Expression and Evolution of Short-Chain Dehydrogenases/Reductases in Nile Tilapia (*Oreochromis niloticus*)

**DOI:** 10.3390/ijms22084201

**Published:** 2021-04-18

**Authors:** Shuai Zhang, Lang Xie, Shuqing Zheng, Baoyue Lu, Wenjing Tao, Xiaoshuang Wang, Thomas D Kocher, Linyan Zhou, Deshou Wang

**Affiliations:** 1 Key Laboratory of Freshwater Fish Reproduction and Development (Ministry of Education), Key Laboratory of Aquatic Science of Chongqing, School of Life Sciences, Southwest University, Chongqing 400715, China; zs1011487839@163.com (S.Z.); memcpy@email.swu.edu.cn (L.X.); zhengsq0825@163.com (S.Z.); lby12530@163.com (B.L.); enderwin@163.com (W.T.); wxs958386@163.com (X.W.); 2 Department of Biology, University of Maryland, College Park, MD 20742, USA; tdk@umd.edu

**Keywords:** short-chain dehydrogenases/reductases superfamily, evolution, expression, Nile tilapia

## Abstract

The short-chain dehydrogenases/reductases (SDR) superfamily is involved in multiple physiological processes. In this study, genome-wide identification and comprehensive analysis of *SDR* superfamily were carried out in 29 animal species based on the latest genome databases. Overall, the number of *SDR* genes in animals increased with whole genome duplication (WGD), suggesting the expansion of *SDR*s during evolution, especially in 3R-WGD and polyploidization of teleosts. Phylogenetic analysis indicated that vertebrates SDRs were clustered into five categories: classical, extended, undefined, atypical, and complex. Moreover, tandem duplication of *hpgd-a, rdh8b* and *dhrs13* was observed in teleosts analyzed. Additionally, tandem duplications of *dhrs11-a*, *dhrs7a*, *hsd11b1b,* and *cbr1-a* were observed in all cichlids analyzed, and tandem duplication of *rdh10-b* was observed in tilapiines. Transcriptome analysis of adult fish revealed that 93 *SDR*s were expressed in more than one tissue and 5 in one tissue only. Transcriptome analysis of gonads from different developmental stages showed that expression of 17 *SDR*s were sexually dimorphic with 11 higher in ovary and 6 higher in testis. The sexually dimorphic expressions of these *SDRs* were confirmed by in situ hybridization (*ISH*) and qPCR, indicating their possible roles in steroidogenesis and gonadal differentiation. Taken together, the identification and the expression data obtained in this study contribute to a better understanding of SDR superfamily evolution and functions in teleosts.

## 1. Introduction

Short-chain dehydrogenases/reductases (SDR) superfamily comprises NAD(P)(H)-dependent oxidoreductases that possess the conserved Rossmann-fold motif. They are a large and ancient gene family found in organisms as diverse as viruses and vertebrates [1].With the rapid release of genome databases for numerous species, the size of the *SDR* superfamily has grown considerably since the initial family members were characterized in the late 1970′s [2,3,4,5]. Genome-wide identification of *SDR*s was performed in cyanobacteria [6], nematode and fruit fly [7], rat, mouse, and human [5,8,9], and plants [10]. Generally, *SDR*s are divided into seven different categories termed “classical”, “extended”, “intermediate”, “divergent”, “complex”, “atypical”, and “undefined” according to their different cofactor binding sites, substrate binding sites, functions, and sequence structures [1]. However, understanding the evolutionary relationships among the *SDR* superfamily genes is difficult because most family members share only 20–30% amino acid sequence similarity.

The diversification and the morphological innovations of vertebrates are attributed to large-scale gene or genome duplications at the origin of the group. These duplications are predicted to have occurred in two rounds of genome duplication, the “2R” hypothesis, or in one genome duplication plus many segmental duplications, although these hypotheses are still somewhat controversial [11]. In addition, teleost fish underwent three round of genome duplication (3R-WGD) [12]. Some teleosts such as rainbow trout (*Oncorhynchus mykiss*) and Atlantic salmon (*Salmo salar*) underwent an additional round of genome duplication (4R) [13,14]. Therefore, teleost fish represent a unique model for studies on evolution. Although fish represent the largest and the most diverse group of vertebrates, comprehensive identification and genome-wide study on the *SDR* superfamily are still lacking. The available genome sequences of more and more species provided new resources to understand the evolution of SDR superfamily. Recently, genome sequences were released for elephant shark (*Callorhinchus milii,* a chondrichthyan) [15], coelacanth (*Latimeria menadoensis,* an early sarcopterygian) [16], and spotted gar (*Lepisosteus productus*, a non-teleost actinopterygian) [17], which represent the key nodes for studying WGD in vertebrates. Comparative genomic analysis and gene mapping of *SDR* superfamily in vertebrates provide insights into the evolution of this gene family after each round of WGDs.

Nowadays, identification and characterization of some subfamily of SDR superfamily are reported in several vertebrates. Genome wide identification and expression analysis of beta-hydroxysteroid dehydrogenase genes were reported in orange-spotted groupers (*Epinephelus coioides*) [18]. Upregulation and downregulation of beta-hydroxysteroid dehydrogenase genes are found in both brain and gonads during sex reversal, indicating their critical roles in neurogenesis of brain and sexual maintenance in orange spotted groupers [19]. In humans, at least 14 separate enzymes that all display catalytic activities towards 17β-hydroxy and keto-steroid substrates were identified [20,21]. In olive flounder (*Paralichthys olivaceus*), eight *hsd17b* family genes, including *hsd17b3*, -*4*, -*7*, -*8*, -*10*, -*12a*, -*12b*, and -*14*, were identified. Flounder *hsd17b10*, -*12a,* and -*12b* are highly expressed in the ovary, while *hsd17b3* is dominantly expressed in the testis. In mammalian species, 11β-hydroxysteroid dehydrogenases (*HSD11b1* and *HSD11b2*) catalyze the interconversion between active and inactive glucocorticoid [21]. In contrast, fish *hsd11b1* proved to be a potent enzyme for the biosynthesis of 11-ketotestosterone (11-KT) from testosterone in testis [22]. Previous study revealed that *dhrs11* can act as an NADPH-dependent *hsd17b* and play an important role in the metabolism of 11-oxygenated-C19-steroids as well as estrogens, androgens, and androgen precursors [23,24]. *Rdh* is another well-defined large subfamily of SDR which is involved in the first step of retinoic acid (RA) synthesis from vitamin A by catalyzing the oxidation of retinol to retinaldehyde [25]. However, these studies have thus far been restricted to expression and function data for a single gene or a subcluster of SDR superfamily in limited tissues or at limited stages of development [24,26]. The overall expression profiles of the *SDR*s in different tissues and at different stages of gonadal development are largely unknown in vertebrates, especially in teleosts.

The Nile tilapia (*Oreochromis niloticus*) is a commercially important farmed fish species in aquaculture worldwide. It is also a good model for study of sex determination and differentiation for the availability of genome sequences, mono-sex fry, and transcriptome data from different tissues [27] and gonads from different developmental stages [28,29]. Given the versatility and the fundamental importance of SDRs in lipid, amino acid, carbohydrate, hormone, and xenobiotic metabolism as well as in redox sensor mechanisms, we carried out an overall identification and comprehensive analysis of the chromosome location, phylogeny, synteny, and spatiotemporal expression profiles in Nile tilapia. Our results provide new insights into the evolution of SDRs and reveal their potential roles in vertebrates.

## 2. Results

### 2.1. Identification of SDRs from Nile tilapia and Representative Species

**Figure 1:**
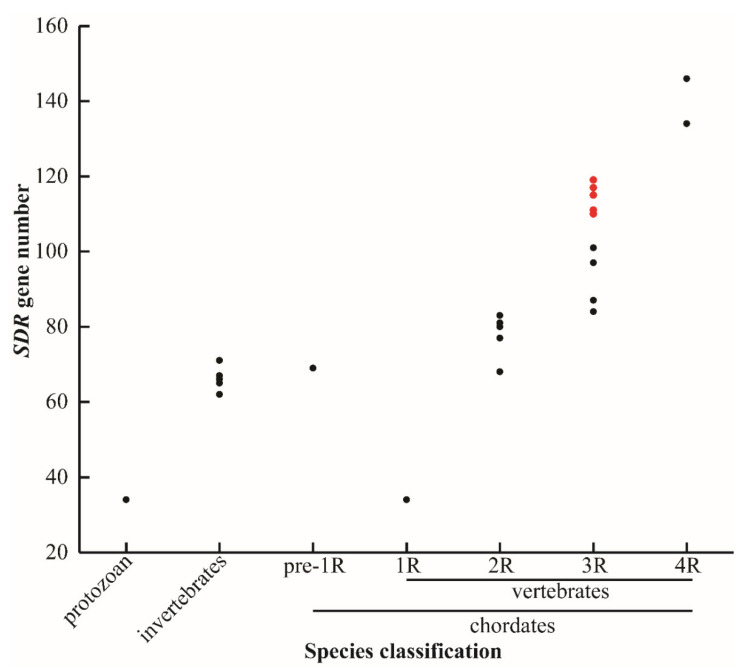
*SDR* gene numbers in different species. Protozoan represents unicellular animals: paramecium (34). Invertebrates: fruit fly (62), domestic silkworm (65), nematode (66), sponge (67), and black tiger shrimp (71). Pre-1R species: vase tunicate (69). 1R species: lamprey (34). 2R species: elephant shark (68), human (77), spotted gar (80), coelacanth (80), tropical clawed frog (81), chicken (83), and python (83). 3R species: channel catfish (84), zebrafish (87), medaka (97), fugu (97), large yellow croaker (101), zebra mbuna (114), Flier cichlid (111), Eastern happy (117), blue tilapia (110), Mozambique tilapia (114), blackchin tilapia (115), and Nile tilapia (119). 4R species: common carp (134) and rainbow trout (146). Red dots represent seven cichlids. 1R, 2R, 3R, and 4R represent four rounds of whole genome duplication (WGD). The identified *SDR*s in lamprey is extraordinarily low, probably due to poor genome assembly.

In total, 119 *SDR*s were identified in the Nile tilapia genome. Of them, nine *SDR*s including *bdh1*, *c-factor*, *hsd17b7*, *rdh12*, *rdh13*, *blvrb*, *ak7*, *dhrsx,* and *hsd11b1* had two copies. In addition, some *SDR*s had multiple copies, for example, three replicates for *hsd17b12*, *dhrs13*, *rdh14*, *cbr1*, *dhrs12,* and *rdh10*, four replicates for *rdh8*, *rdh12,* and *hpgd*, and five replicates for *dhrs7*. Interestingly, 14 replicates of *dhrs11* were isolated from Nile tilapia. Additionally, *SDR*s were isolated from other 28 animal species with different numbers of WGD (Figure 1). The sequences of *SDR*s in blue tilapia, Mozambique tilapia, and blackchin tilapia were identified from their genomes that were sequenced by our lab and are shown in Supplementary file 1. The *SDR* gene name, the accession number, and the chromosome location for all other species are listed in Tables S1–S26. The total number of *SDR*s changed remarkably in species that experienced 3R- and 4R-WGD, while it remained basically constant in protozoan, invertebrates, and species that experienced 1R- and 2R-WGD. It is noteworthy that there is a great difference in *SDR* numbers between cichlids and other teleost species.

**Figure 2:**
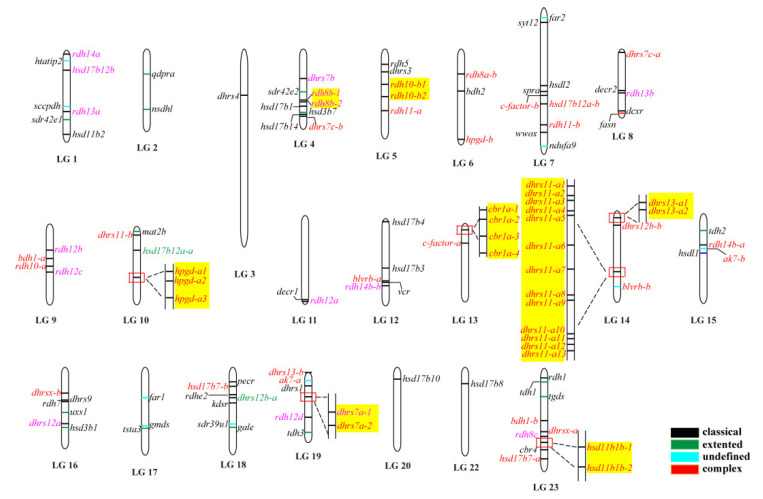
Chromosomal maps of the *SDR*s in Nile tilapia. *SDR*s were depicted on linkage group (LG) of the genome. LG21 is absent because it was combined with LG16. Black, green, blue, and red horizontal lines indicate classical, extended, atypical, and complex *SDR*s, respectively. Gene names in pink and red indicate genes derived from 2R and 3R events, respectively, whereas gene names in green represent it undergoing both 2R and 3R events. Gene name in black is a single copy gene. Tandem duplicates are highlighted in a yellow shade.

The 119 *SDR*s were unevenly distributed across all of the 22 linkage groups of Nile tilapia. For instance, there were 17 *SDR*s located on LG14, while there was only one on LG3, LG20, and LG22. Eight *SDR*s genes (*rdh8*, *rdh10*, *hpgd*, *cbr1*, *dhrs7*, *dhrs11*, *dhrs13,* and *hsd11b1*) had multiple copies located together in tandem arrays. These tandemly arrayed genes were on different chromosomes, except *dhrs11* and *dhrs13,* which were located on LG14 (Figure 2). In total, eight *SDR*s (*dhrs7a*, *dhrs11-a*, *dhrs13-a*, *rdh8b*, *rdh10-b*, *hpgd-b*, *cbr1a,* and *hsd11b1b*) in Nile tilapia went through tandem duplication, which resulted in 2, 13, 2, 2, 2, 3, 4, and 2 copies, distributed on LG19, LG14, LG14, LG4, LG5, LG10, LG13, and LG23, respectively.

### 2.2. Phylogenetic and Syntenic Analyses of SDRs

**Figure 3:**
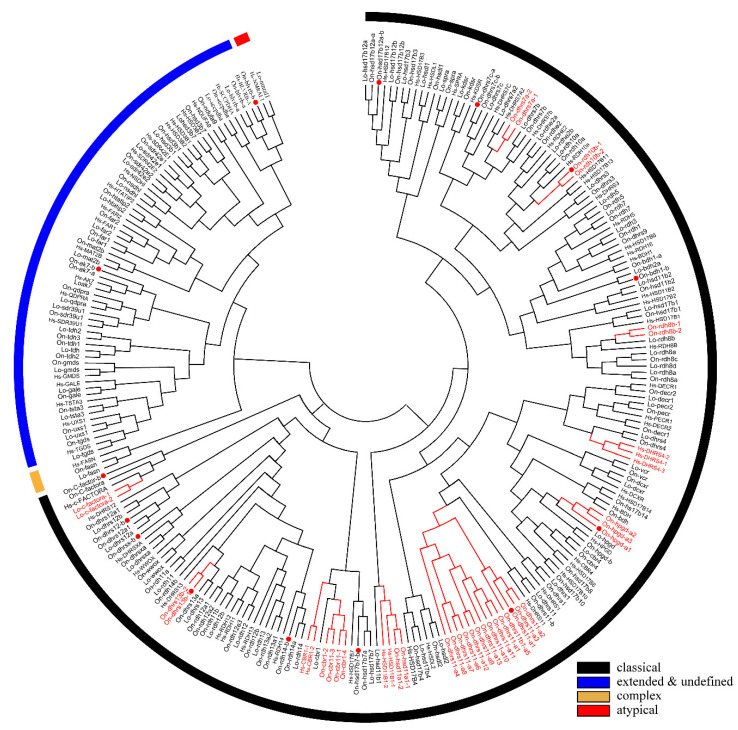
Phylogenetic tree of Nile tilapia, human, and spotted gar *SDR* family. The NJ method was used to construct the tree by MEGA 6.0. The multiple alignment software Bioedit was used to align the amino acid sequences of the conserved domain. On, *Oreochromis niloticus*; Lo, *Lepisosteus oculatus;* Hs, *Homo sapiens*. Three different colored arcs on the outside represent different categories of *SDR*s. The red lines and the gene names represent genes with tandem duplications. The red dots represent genes stemmed from 3R. Two copies of *cbr1* were observed in zebrafish and channel catfish and one copy in Nile tilapia, therefore, the 3R-event of this gene was presented in Figure S2. GenBank accession numbers of each sequence used are listed in Tables S4, S12, and S15.

A phylogenetic tree was constructed using the conserved domain of amino acid sequences of all SDRs from Nile tilapia, spotted gar, and human. The SDRs were divided into five categories termed “classical”, “undefined”, “extended”, “complex”, and “atypical”. Most of the members belonged to the “classical” category. Only one member of the “complex” category was observed in human, spotted gar, and Nile tilapia, respectively. One member of “atypical” category was observed in human and spotted gar but none in Nile tilapia. “Classical”, “atypical”, and “complex” SDRs were clustered into different clades, while “extended” and “undefined” SDRs were clustered in one clade due to their high sequence similarity (Figure 3). “Intermediate” and “divergent” genes were not identified in the study. Among the 119 *SDR* members of Nile tilapia, 114 genes could be designated into 49 subfamilies according to previous publications [5]. Compared with human, Nile tilapia has two additional subfamilies, SDR112c and SDR348c, but lacks SDR48a. Subfamily designations of *SDR* genes in Nile tilapia, spotted gar and human are listed in Table S27.

**Figure 4:**
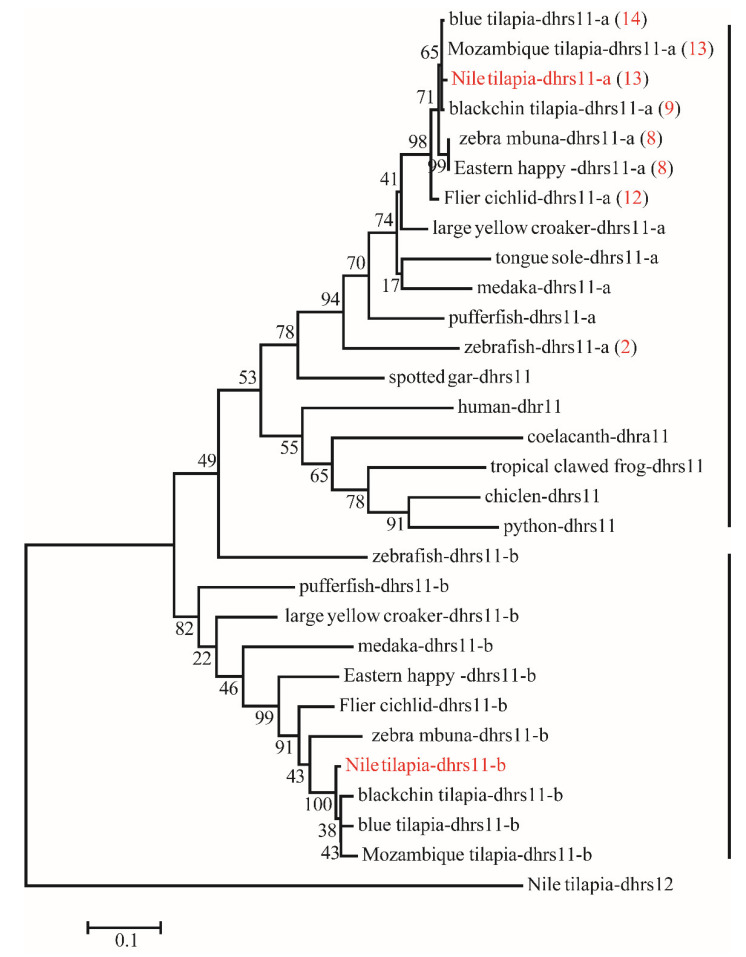
Phylogenetic tree of *dhrs11* in vertebrates. The ML tree was constructed using the amino acid sequences from different species using Nile tilapia *dhrs12* as the outgroup. All sequences of *dhrs11* were clustered into two branches termed *dhrs11-a* and *dhrs11-b*. The red numbers in brackets represent the number of tandem duplicates in corresponding species. Numbers at the branch of the phylogenetic tree stand for bootstrap.

Phylogenetic analyses revealed that duplicates of 15 *SDR*s (*bdh1*, *c-factor*, *cbr1*, *dhrsx*, *hsd17b7*, *rdh12*, *blvrb*, *dhrs11*, *dhrs13*, *dhrs12b*, *dhrs7c*, *rdh14b*, *hsd17b12a, hpgd,* and *ak7*) were retained after the 3R-WGD events (Figure 3). Further phylogenetic analyses revealed that duplicates of four *SDR*s (*dhrs12*, *hsd11b1*, *rdh14,* and *hsd17b12*) were retained after the 2R-WGD (Figure S1). *cbr1* retained two copies in zebrafish and channel catfish, suggesting one of them was retained after 3R-WGD, and *rdh8* had multiple copies in lower species they retained since early replication (Figure S2). In addition, phylogenetic trees of six genes (*bdh1*, *c-factor*, *dhrsx*, *hsd17b7*, *blvrb,* and *ak7*), which retained two copies and had no tandem duplicates, were constructed (Figure S3). Moreover, eight genes (*dhrs11*, *rdh8*, *rdh10*, *hpgd*, *cbr1*, *dhrs7*, *hsd11b1,* and *dhrs13*) were tandem duplicated in different species, which resulted in multiple copies on the same chromosome (Figure 3 and Figure 4, and Figure S4). Among these genes, *hpgd-a*, *rdh8b,* and *dhrs13-b* were tandem duplicated in teleosts, *dhrs11-a*, *dhrs7a*, *hsd11b1-b,* and *cbr1-a* were tandem duplicated in cichlids, and *rdh10-b* was tandem duplicated in tilapiines. A phylogenetic tree of *dhrs11* in vertebrates demonstrated a duplication of *dhrs11* corresponding to the teleost-specific 3R-WGD, and 8 to 14 tandem duplicates of *dhrs11-a* were observed in cichlids. Interestingly, two copies of *dhrs13* were observed on adjacent positions of the same chromosome in all bony fishes, indicating that tandem duplication of *dhrs13* occurred in their ancestor. In addition, species specific tandem duplication of some *SDR* genes was observed in some vertebrates, such as *hsd11b1b* in elephant shark and coelacanth (Figure S1, and *cbr1-a* in human (Figure S2).

**Figure 5:**
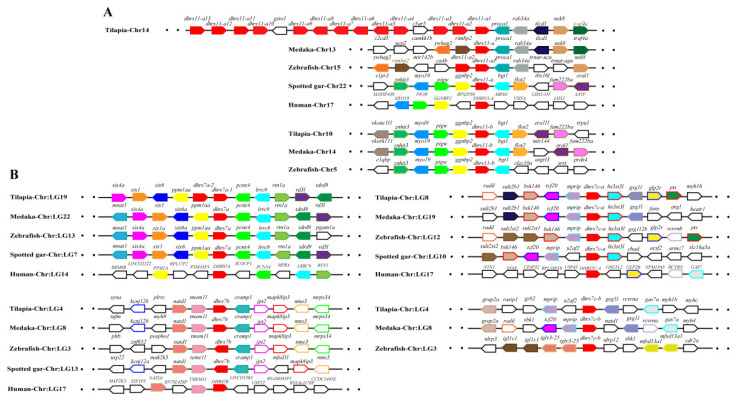
Syntenic analysis of *dhrs11* (**A**) and *dhrs7* (**B**) and their adjacent genes in Nile tilapia and other vertebrates. Rectangles represent genes in chromosome/scaffold. Dots represent omitted genes of the chromosome/scaffold. The direction of the arrows indicates the gene orientation. The *SDR*s are shown in red, while the other genes are shown in different colors.

Synteny analysis was performed for duplicated *SDR* genes to further conform their origin. Duplicates of *dhrs12*, *rdh14*, *hsd11b1,* and *hsd17b12* were derived from 2R-WGD, as the synteny was shared in vertebrates (Figure S5), while duplicates of *bdh1*, *c-factor*, *cbr1*, *dhrsx*, *hsd17b7*, *rdh12*, *blvrb*, *dhrs11*, *dhrs13*, *dhrs12b*, *dhrs7c*, *rdh14b*, *hsd17b12a, hpgd,* and *ak7* were derived from 3R-WGD, as the synteny was conserved only in teleosts (Figure 5, Figures S5 and S6). In addition, tandem duplication was observed for *dhrs7*, *dhrs11*, *dhrs13*, *hpgd*, *hsd11b1,* and *rdh10*. Synteny analysis also revealed tandem duplication for *cbr1* and *rdh8* (Figure S7). Conserved synteny of *dhrs11-b* and its upstream genes (*myo19*, *pigw,* and *ggnbp2*) and downstream genes (*big2*, *fam22ba,* and *cra11*) were observed in Nile tilapia, zebrafish, and medaka. Similarly, conserved synteny of *dhrs11-a* and its upstream genes (*tlcd1* and traf4a) and its downstream genes (*ywhag2*, *nek8*, *rimbp2*, *procal*) were observed in Nile tilapia, zebrafish, and medaka but not in non-teleost vertebrates. Interestingly, 14 copies of *dhrs11* genes were found in Nile tilapia, while there were only two copies in most teleosts and three copies in zebrafish (Figure 5A). Conserved synteny of three *dhrs7*, named as *dhrs7a, dhrs7b,* and *dhrs7c*, was observed throughout vertebrates, and *dhrs7c* was further duplicated in teleosts, as reflected by conserved synteny of *dhrs7c-a* and *dhrs7c–b* with their adjacent genes. In contrast, tandem duplication of *dhrs7a* (*dhrs7a-1* and *dhrs7a-2*) was found in Nile tilapia (Figure 5B). These synteny analyses provide strong evidence for determining the origin of different genes.

### 2.3. Tissue Distribution and Ontogenic Expression of SDRs in Nile Tilapia Gonads

**Figure 6:**
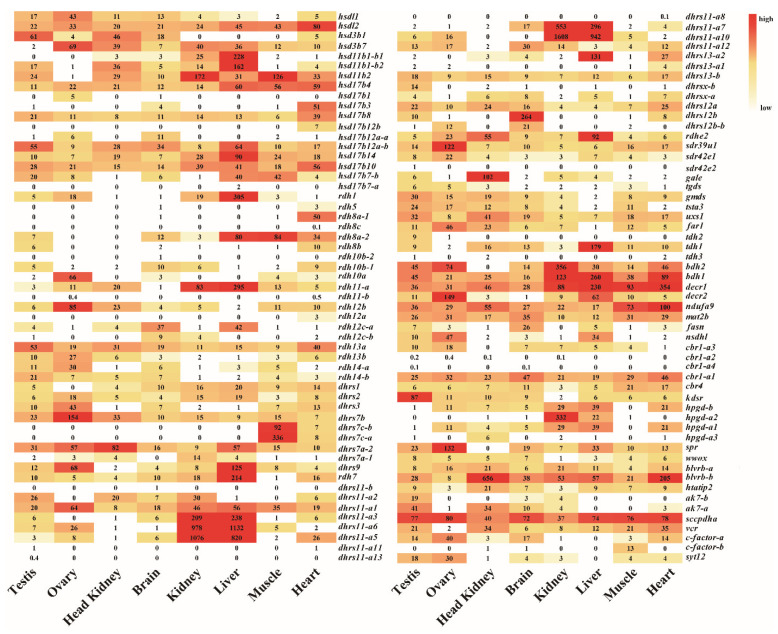
The expression profiles (RPKM) of *SDR*s in eight tissues based on transcriptome data in Nile tilapia. Color gradients indicate differences in expression levels. Each row represents a different gene, and each column represents an independent tissue sample. The widespread complex expression patterns in all tissues were readily discernable.

Transcriptome data from eight adult tissues and gonads from four developmental stages of Nile tilapia allowed us to analyze the spatial and the temporal expression profiles of *SDR*s (Figure 6). There were 92 *SDR*s expressed in at least one tissue with 85 genes in the brain, 88 genes in the heart, 74 genes in the kidney, 73 genes in the liver, 85 genes in the testis, 73 genes in the ovary, 78 genes in the muscle, and 68 genes in the head kidney. In total, 35 genes were ubiquitously expressed in all 8 tissues, while 18 genes were expressed at background levels. It is noteworthy that *dhrs12a*, *decr1*, *dhrs11-a10*, *dhrs11-a6*, *kdsr*, *decr2*, *dhrs7*c-a, and *blverb-b* showed the highest expression in brain, heart, kidney, liver, testicle, ovary, muscle, and head kidney, respectively. The two *dhrs7c* (*dhrs7c-a* and *dhrs7c-b*) were specifically expressed in the muscle, while *gale* was expressed only in the head kidney. Moreover, ten *SDR*s (*rdhe2*, *hsd11b1a-1*, *hsd11b1b-2, hsd17b14*, *rdh1*, *dhrs9*, *rdh11a*, *rdh7-a*, *dhrs13-b2,* and *tdh1*) were predominantly expressed in the liver. Importantly, *hsd3b1* and *kdsr* were exclusively expressed in the testis, while *hsd3b7*, *rdh12b*, *decr2*, *dhrs7b*, *sdr39u1,* and *spra* were dominantly expressed in the ovary. Seven copies of the 13 *dhrs11-a* were highly expressed in both liver and kidney.

Analysis of the gonadal transcriptomic data from four critical development stages revealed that 95 *SDR*s were expressed in Nile tilapia gonads according to the threshold we set, while 56 *SDR*s were highly expressed (total RPKM > 100). The expression of *SDR*s (RPKM) in Nile tilapia gonads at four developmental stages was listed Table S28. Based on the transcriptome data, 17 genes displayed high and sexually dimorphic expression in adult gonads. The expression levels of 11 ovary-enriched genes (*hsd3b7*, *rdh10a*, *rdh12b*, *dhrs3*, *dhrs7b*, *dhrs9 sdr39u1*, *hpgd-b2*, *far1*, *spra*, *decr2*) were found to peak at 90 dah, followed by 180, 30, and 5 dah. Expression levels of six testis-enriched genes (*hsd3b1*, *hsd11b1-b2*, *hsd11b2*, *rdh14b*, *kdsr,* and *ak7-a*) displayed similar expression profiles as the ovary-enriched genes but with higher expression in XY than in XX gonads (Figure S8).

### 2.4. Validation of the Transcriptome Expression Profile by ISH and qPCR

**Figure 7:**
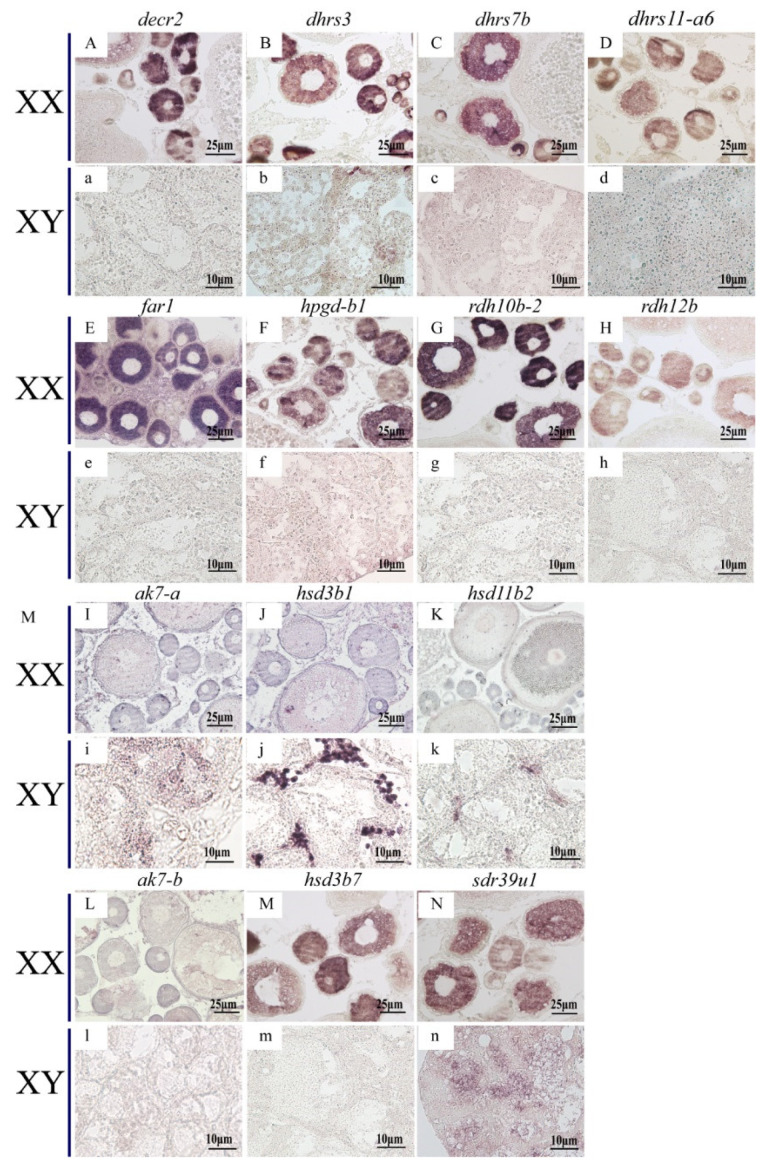
Cellular localization of 14 *SDR*s in the Nile tilapia gonads at 180 dah by *in situ* hybridization. Nine of them were expressed exclusively in the cytoplasm of phase **I** and II oocytes (**A–H**,**M**), but not expressed in testis (**a–h**,**m**)*. ak7-b* was not expressed in neither ovary nor testis (**L**,**l**). *sdr39u1* was expressed in phase II oocytes of ovary (**N**) and spermatocytes of testis (**n**). The other three *SDR*s were expressed in the Leydig cells of testis exclusively (**i**–**k**), but not expressed in ovary (**I**–**K**).

**Figure 8:**
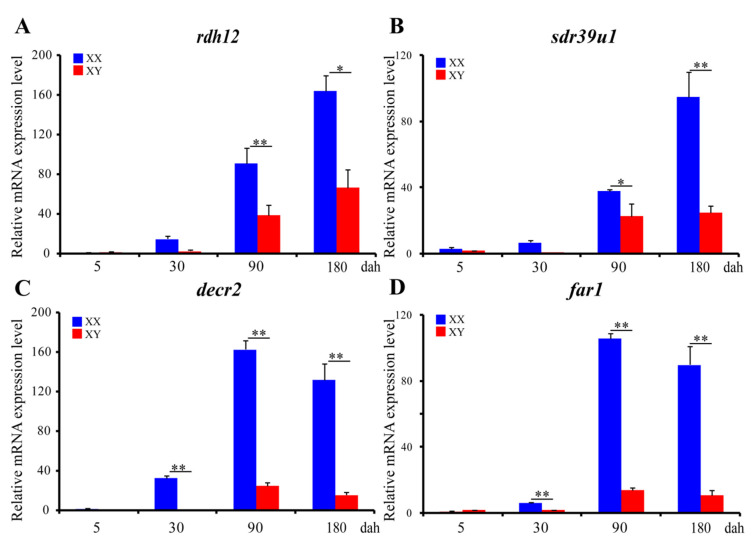
Ontogeny expression of *rdh12* (**A**), *sdr39u1* (**B**), *decr2* (**C**), and *far1* (**D**) in gonads at 5, 30, 90, and 180 dah analyzed by qPCR (n = 3). *eef1a1a* was used as internal control. Data were expressed as the mean ± SD. “*” and “**” above the error bar indicate statistically significant differences between XX and XY gonad at *p* < 0.05 and *p*< 0.01 by Student’s *t-*test, respectively. All examined genes displayed similar expression profiles to those from the transcriptome data.

*ISH* was performed to detect the cellular localization of the selected 14 *SDR*s (*decr2*, *dhrs3*, *dhrs7b*, *dhrs11-a6*, *far1*, *hpgd-b1*, *hsd3b1*, *ak7-a*, *ak7-b*, *hsd3b7*, *hsd11b2*, *rdh10b-2*, *rdh12b*, *sdr39u1*) in gonads of Nile tilapia at 180 dah. Nine (*decr2*, *dhrs3*, *dhrs7b*, *dhrs11-a6*, *far1*, *hpgd-b1*, *rdh10b-2*, *rdh12b,* and *hsd3b7*) of them were expressed dominantly in the cytoplasm of phase I and II oocytes in the ovary. On the contrary, *hsd3b1* and *hsd11b2* were expressed in the Leydig cells, and *ak7-a* was expressed in somatic cells of the testis. Expression of *sdr39u1* was detected in both the spermatocytes of the testis and the cytoplasm of phase II oocytes in the ovary (Figure 7). Additionally, qPCR was performed to validate the transcriptome data of *rdh12b*, *sdr39u1*, *far1,* and *decr2*. Consistently, very low or background expression level was detected for the four genes at 5 dah. The expressions of *rdh12b* and *sdr39u1* displayed no significant differences between XX and XY gonad at 30 dah, but expressions of both genes were significantly higher in XX ovary than in XY testis at 90 and 180 dah, while the expressions of *decr2* and *far1* were significantly higher in XX ovary than in XY testis at 30, 90, and 180 dah (Figure 8). Primers used for *ISH* and qPCR in this study are listed in Table S29.

## 3. Discussion

SDR enzymes play critical roles in various physiological and metabolic processes from archaea and bacteria to eukaryotes. However, the genome-wide evolution, expression, and function of the SDR superfamily in the animal kingdom, especially in teleosts, has yet to be elucidated. In the present study, we identified and performed phylogenetic and syntenic analyses of *SDR*s from the genomes of representative vertebrates and invertebrates. We then quantified the expression of Nile tilapia *SDR*s in different adult tissues and gonads from four critical development stages.

### 3.1. Identification, Phylogenetic, and Syntenic Analyses of SDRs

Studies on *SDR* superfamily mainly focused on the molecular structure, the substrates, and the catalytic property of a single gene or different subclusters. Thus far, genome wide identification of *SDR* superfamily has been done in a few species, including cyanobacteria [6], nematode and fruit fly [7], plants [10], and humans [30]. Overall identification and expression analyses of *SDR*s members are of great importance to define their diverse biological functions. Fish, being the largest group of vertebrates with more than 30,000 species, diverse habitats, reproductive patterns, and strong environmental adaptability, occupy an important position in evolution [31]. In this study, we isolated *SDR*s from the genomes of 29 animal species, including Nile tilapia. Most *SDR*s in Nile tilapia belong to the “classical” category, followed by the “extended” category, which is consistent with the results reported for human [5]. In contrast, both “complex” and “atypical” categories have one member only. Among the 119 *SDR*s of Nile tilapia, 114 members were assigned into 49 subfamilies, while the classifications of the remaining 5 genes were uncertain. SDR48A, which also exists in human genome, was not detected in Nile tilapia. In contrast, two subfamilies, SDR112C and SDR348C, which are absent in human genome, were identified in Nile tilapia genome, with one *SDR* for each subfamily. Gene duplication is assumed to have played a crucial role in the evolution of vertebrates. Four rounds of large-scale genome duplications (referred to as 1R, 2R, 3R, and 4R) shaped genome evolution in fish. In combination with phylogenetic and syntenic analyses, we concluded that *SDR* superfamily members increased evidently due to the 3R- and the 4R-WGD in fish. Although teleost fish experienced the 3R-WGD, the extended *SDR*s has not expanded much, indicating that a large number of the extended *SDR* subfamilies in teleost fishes were lost during evolution. Variation in the number of *SDR* superfamily in different representative species is consistent with multiple WGD events. Tandem duplications of *dhrs11-a*, *hsd11b1b*, *cbr1-a,* and *dhrs7a* were observed in seven cichlids analyzed, and tandem duplication of *rdh10-b* was only observed in four tilapiine species, indicating that they are lineage specific. In addition, tandem duplications of *SDR*s were also observed in other species, such as *far*, *hsd11b1,* and *dhrs4* in humans and *c-factor* in spotted gar. These results demonstrated that tandem duplication is common in the *SDR* superfamily in vertebrates. The different number of *SDR*s observed in teleosts might be mainly attributed to different tandem duplication of different *SDR* genes among them. It is worth noting that some *SDR* genes have multiple copies located on different chromosomes in some invertebrates. These duplicates probably resulted from transposon-mediated replication. The origin of these copies remains to be elucidated.

### 3.2. Spatial and Temporal Expression of SDRs in Nile Tilapia

SDR superfamily encoded a large number of enzymes and displayed a broad spectrum of metabolic functions [1]. Transcriptome data revealed that the majority of *SDR*s are expressed in multiple tissues, indicating that SDRs may play roles in various physiological and metabolic processes in Nile tilapia. In humans, *DHRS7* is expressed in prostate, adrenal glands, liver, and intestine and participates in the reductive metabolism of both steroids and retinoids [32]. Over-expression of SRP-35 (*DHRS7C*) in mouse skeletal muscles induces enhanced glucose metabolism [33]. In the present study, five *dhrs7*, *dhrs7a-1*, *dhrs7a-2*, *dhrs7b*, *dhrs7c-a,* and *dhrs7c-b*, were identified in Nile tilapia. *Dhrs7b* was expressed in ovary, indicating its possible role in steroid biosynthesis. *Dhrs7c-a* and *dhrs7c-b*, which originated from fish specific 3R-WGD, were highly expressed in muscle, suggesting their possible role in glucose metabolism. Previous reports demonstrated that human BLVRB has several biochemical functions, including biliverdin and riboflavin reductase (NADPH) activity. It catalyzes the reduction of biliverdin tetrapyrrole as an intermediary redox substrate in bilirubin generation in the heme degradation pathway [34]. In Nile tilapia, high levels of expression of *blvrb* were detected in both heart and head kidney, suggesting their possible role in heme metabolism. Human DHRS11 exhibits enzymatic activities of both 17β-hydroxysteroid dehydrogenase and 3β-hydroxysteroid dehydrogenase, which efficiently catalyze the reduction of 11KA4, 11K-Adione, and 11KAST [23,35]. The tandem duplication of *dhrs11* in cichlids and the high expression of different copies in different tissues suggested their importance in multiple functions. High expression of *dhrs11-a5*, *dhrs11-a6,* and *dhrs11-a10* in both kidney and liver indicated their essential roles in metabolism and homeostasis in Nile tilapia. In zebrafish, *dhrs12* (named as 36K) was identified in myelin of the central nervous system to regulate membrane lipid composition and influence oligodendrocyte precursor cell differentiation and further myelination by altering the amount of transmembrane Notch ligands [36]. Consistently, *dhrs12a* gene was found to be expressed exclusively in Nile tilapia brain, indicating its critical role in brain functions.

It is well documented that sex steroids play essential roles in fish sex differentiation, gametogenesis, and reproduction [37,38]. Recent studies showed that RA is critical for initiation of meiosis in both mammals and teleosts [39,40]. It is well known that SDRs play important roles in steroids and retinoids metabolism [25]. Expression, substrate specificity, and functions of *hsd17b*, *hsd3b*, and *hsd11b* in sex steroids biosynthesis were extensively investigated in several teleosts [41,42,43,44]. In this study, *hsd3b7*, *rdh10b-2*, *rdh12b*, *dhrs3*, *dhrs7b*, *dhrs9*, *sdr39u1*, *hpgd-b1*, *far1*, *spra,* and *decr2* were identified as ovary-enriched genes, while *hsd3b1*, *hsd11b1-b2*, *hsd11b2*, *rdh14b*, *kdsr,* and *ak7-a* were identified as testis-enriched genes. Sexually dimorphic expression of *SDR*s in Nile tilapia gonads indicated their essential roles in steroids and RA metabolism. However, the exact role of the newly identified ovary- and testis-enriched *SDR* genes remains to be elucidated. Further investigations of the functions of these *SDR*s in fish gonads will greatly promote the understanding of their roles in sex steroids and RA metabolism.

Transcriptome analyses revealed that all of the ovary-enriched genes and the testis-enriched genes were highly expressed at 90 and 180 dah, indicating that they might be important for oogenesis and spermatogenesis, respectively. *rdh12b*, *sdr39u1*, *decr2*, and *far1* were significantly up-regulated from 90 dah in females, indicating their indispensable roles in oogenesis. Previous studies showed that *rdh12b* recognizes both retinoids and lipid peroxidation products (C9 aldehydes) as substrates and contributes to the reduction of all-trans-retinaldehyde [45]. *Decr2* gene encodes the peroxisomal 2,4-dienoyl-CoA reductase, which primarily converts 2,4-dienoyl-CoA to trans-3-enoyl-CoA. Biochemical studies and structural analysis suggest that human DECRs can catalyze the shortening of six-carbon-long substrates and shorten very long chain fatty acids [46,47]. *Far1* catalyzes the reduction of saturated and unsaturated C16 or C18 fatty acyl-CoA to fatty alcohols and plays an essential role in the production of ether lipids/plasmalogens in mammals [48]. These three genes might be responsible for the formation of oil droplets in oocytes in fish, which contain mainly neutral lipids rich in monounsaturated fatty acids, which serve as metabolic energy reserves [48]. In contrast, the role of *sdr39u1* in vertebrates is still largely unknown. Taken together, the abundant expression of *SDR*s in ovary indicated that anabolism of RA and fatty acid is critical for oogenesis in fish.

In fish, both RA and 11-KT proved to play essential roles in testicular differentiation and spermatogenesis. Previous reports showed that both *HSD17b3* and *HSD11b2* might be involved in the biosynthesis of 11-KT in human [41]. *RDH14* [49] is a ubiquitously expressed microsomal enzyme, which might function as a reductase and contribute to the reduction of retinaldehyde to retinol in most human tissues. The abundant expression of *rdh14* in testis of Nile tilapia further emphasizes the essentiality of retinol for testicular differentiation and functions. In humans, 11HSD1, which is ubiquitously expressed in most tissues, catalyzes cortisone to cortisol, while 11HSD2, expressed in the kidney and other classical target tissues for aldosterone, is responsible for the inactivation of cortisol to cortisone [50]. In fish, the recombinant Hsd11b, with 11β-dehydrogenase activity metabolizing from cortisol to cortisone and 11β-hydroxytestosterone to 11-KT, is involved in mitosis of spermatogonia and spermatogenesis [41]. In this study, dominant expression of both *11hsdb1*-*a1*, -*a2* and *11hsdb2* was found in testis, suggesting their possible roles in 11-KT production.

## 4. Materials and Methods

### 4.1. Animal Rearing

Nile tilapia fish used in this study were reared in recirculating freshwater tanks at 26 °C and under natural photoperiod. All female (XX) progenies were obtained by crossing the normal female (XX) with the sex-reversed XX pseudomales. All male (XY) progenies were obtained by crossing the normal female (XX) with YY supermales. Animal experiments were performed following the regulations of the Guide for Care and approved by the Institutional Animal Care and Use Committee of Southwest University.

### 4.2. Identification and Nomenclature of the SDRs

*SDR* sequences of human (*Homo sapiens*) and zebrafish (*Danio rerio*) were used as the query sequences to blast the genome sequences by tblastn (E = 2 × 10^−5^)) to identify the *SDR* genes in each genome analyzed. The identified *SDR*s were used to search against the NCBI (https://www.ncbi.nlm.nih.gov/, accessed on 5 May 2020) by blastx to reduce redundant matches. In addition, *SDR* sequences of the rainbow trout (*Oncorhynchus mykiss*), common carp (*Cyprinus carpio*), Nile tilapia (*Oreochromis niloticus*), zebra mbuna (*Maylandia zebra*), Flier cichlid (*Archocentrus centrarchus*), Eastern happy (*Astatotilapia calliptera*), large yellow croaker (*Larimichthys crocea*), medaka (*Oryzias latipes*), fugu (*Takifugu rubripes*), channel catfish (*Ictalurus punctatus*), spotted gar (*Lepisosteus oculatus*), coelacanth (*Latimeria chalumnae*), elephant shark (*Callorhinchus milii*), tropical clawed frog (*Xenopus tropicalis*), chicken (*Gallus gallus*), python (*Python bivittatus*), lamprey (*Lampetra japonicavase*), tunicate (*Ciona intestinalis*), black tiger shrimp (*Penaeus monodon*), domestic silkworm (*Bombyx mori*), fruit fly (*Drosophila melanogaster***)**, nematode (*Caenorhabditis elegans*), sponge (*Amphimedon queenslandica*), and paramecium (*Paramecium tetraurelia***)** were collected from NCBI and Ensembl (http://asia.ensembl.org/index.html, accessed on 18 May 2020) databases. *SDR* sequences of blue tilapia (*Oreochromis aureus*), Mozambique tilapia (*Oreochromis mossambicus*), and blackchin tilapia (*Sarotherodon melanotheron*) were collected from unpublished genome database generated by our lab. Relatively high-quality genome sequences in these species allowed us to isolate more *SDR* members to reflect the true evolutionary history of the superfamily. These 29 species provided a broad evolutionary coverage of different animal groups to test the *SDR* superfamily variation. In order to distinguish genes derived from different rounds of WGD or tandem duplication, we developed a new system of nomenclature by adding numbers or letters as suffixes of the gene name. The gene derived before 3R-WGD was represented by the letters a, b after the gene name. The gene derived from 3R-WGD was represented by a hyphen followed by a, b, etc. The gene produced by tandem duplication was represented by a number after the hyphen or a letter after the hyphen.

### 4.3. Phylogenetic Analysis and Genomic Distribution of SDRs

The amino acid sequences of the conserved domain of SDRs from Nile tilapia, humans, and spotted gar were aligned by Clustal W with default parameters using the multiple alignment software BioEdit (Carlsbad, USA). The neighbor-joining (NJ) method was used for phylogenetic analyses of large numbers of *SDR*s in Nile tilapia, spotted gar, and humans, and the maximum likelihood (ML) method was used for phylogenetic analyses of specific *SDR*s in many different species by MEGA 6.0 software (Tempe, USA) [51]. A bootstrap of 1000 replicates was used to assess the confidence in all phylogenies. *SDR*s from representative species in vertebrates were used in these trees, including those from several species of Cichlidae (*SDR*s from Nile tilapia, zebra mbuna, flier cichlid and eastern happy were downloaded from NCBI, while those from blue tilapia, Mozambique tilapia, and blackchin tilapia were predicted according to their unpublished genome database in our lab. For syntenic analysis, position and orientation of *SDR*s and their adjacent genes on the chromosome were determined using NCBI database.

### 4.4. Expression Analysis of Nile Tilapia SDRs in Adult Tissues and Gonads at Four Critical Developmental Stages

The transcriptomes of eight tissues from adult Nile tilapia, including brain, heart, liver, ovary, testis, kidney, muscle, and head kidney, were downloaded from NCBI database (Accession codes: PRJNA78915 and SRR1916191). RPKM was used to normalize the expression profile of *SDR* genes. Bidirectional hierarchical clustering analyses were performed using the heatmap package [52]. The transcriptomes (accession codes: SRA055700) of four pairs of XX and XY gonads from Nile tilapia at 5, 30, 90, and 180 days after hatching (dah) were downloaded from the NCBI database. The members with total RPKM < 10 in all tissues or RPKM < 1.25 in a single tissue were regarded as the background expression [53].

### 4.5. Validation of Expression Profile of SDRs by qPCR and ISH

The 14 *SDR*s were selected for validation of their gonadal expression at 180 dah by in situ hybridization (*ISH*). Among them, 4 *SDR*s were selected for further validation of expression profile by qPCR. To perform qPCR, gonads dissected from XX and XY Nile tilapia at 5, 30, 90, and 180 dah (about 6–200 fish for each sample depending on the fish size) were collected. The total RNA was isolated from each sample and reverse-transcribed using MMLV reverse transcriptase (Invitrogen, Carlsbad, CA, USA) according to the manufacturer’s protocol. Subsequently, qPCR examination was carried out according to the manufacturer’s protocol of SYBR Green I Master Mix (TaKaRa, Dalian, China). Nile tilapia *eef1a1a* was used as an internal control to normalize the expression of these 4 genes. The relative abundance of mRNA transcripts was evaluated using the formula *R* = 2^−ΔΔCt^. Data were expressed as mean ± SD for triplicates. Statistical analyses were carried out using Student’s *t*-test of the SPSS package, version 18.0.

To ascertain cellular localization of the *SDR* family members in the developing gonads, *ISH* was performed using ovaries and testes from Nile tilapia at 180 dah. Fixation, embedding, and sectioning of dissected gonads and *ISH* were performed as described previously. Probes of sense and antisense digoxigenin (DIG)-labeled RNA strands were transcribed in vitro from linearized pGEM-Teasy-*decr2/dhrs3/dhrs7b/dhrs11-a6/far1/hpgd-b1/ak7-a/ak7-b/hsd3b1/hsd3b7/hsd11b2/rdh10b-2/rdh12b/sdr39u1* DNA using an RNA labeling kit (Roche, Mannheim, Germany).

## 5. Conclusions

In this study, 119 *SDR* members assigned into 49 subfamilies and four categories were identified by comprehensive analyses of genome and transcriptome data in Nile tilapia. Phylogenetic and syntenic analyses demonstrated that tandem duplications in combination with multiple WGDs contributed to the expansion of *SDR* superfamily in vertebrates. In general, the variation of *SDR* numbers reveals the evolution process of *SDR* superfamily. Most *SDR*s were expressed in different tissues, including developing gonads. Some *SDR*s displayed tissue specificity or gender dimorphism. This study provided a new perspective for the evolution of the *SDR* superfamily and laid a solid foundation for revealing the role of the *SDR* genes in bony fishes and even vertebrates, especially in oogenesis and spermatogenesis.

## Data Availability

The data presented in this study are available on request from the corresponding authors.

## Supplementary Materials

All supplementary materials are available online at https://www.mdpi.com/article/10.3390/ijms22084201/s1.

## Abbreviations

SDR	short-chain dehydrogenases/reductases
WGD	whole genome duplication
1R	first round of genome duplication
2R	second round of genome duplication
3R	third round of genome duplication
4R	fourth round of genome duplication
qRT-PCR	quantitative real time polymerase chain reaction
IHC	Immunohistochemistry
NJ	neighbor-joining
dah	day after hatching
RPKM	reads per kb per million reads
