# Supplementary material for: Identification, Expression and Evolution of Short-Chain Dehydrogenases/Reductases in Nile Tilapia (Oreochromis niloticus)
Source: Int J Mol Sci. 2021 Apr 18;22(8):4201. doi: 10.3390/ijms22084201 (PMC8073704; doi:10.3390/ijms22084201)
Supplement: Supplementary file 1 [file ijms-22-04201-s001.zip › Revised Supplemental Figures.pdf]

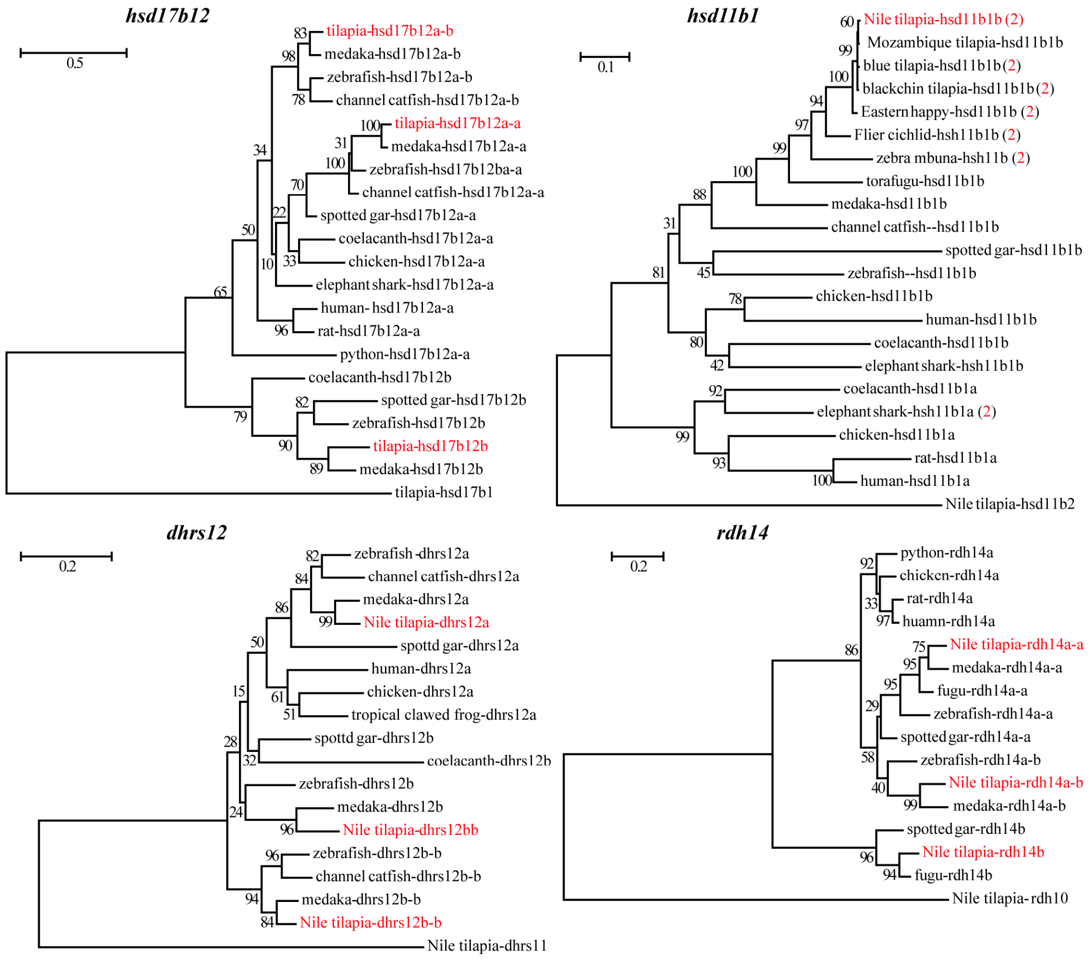

Figure S1 Phylogenetic trees of genes originate from 2R-WGD. The trees were also constructed using their amino acid sequences of different species and ML method. The genes of Nile tilapia were marked with red. GenBank accession numbers of the sequences used are listed in Table S3-S18. Numbers at the branch of the phylogenetic trees stand for bootstrap. Genes were named according to these phylogenetic trees.

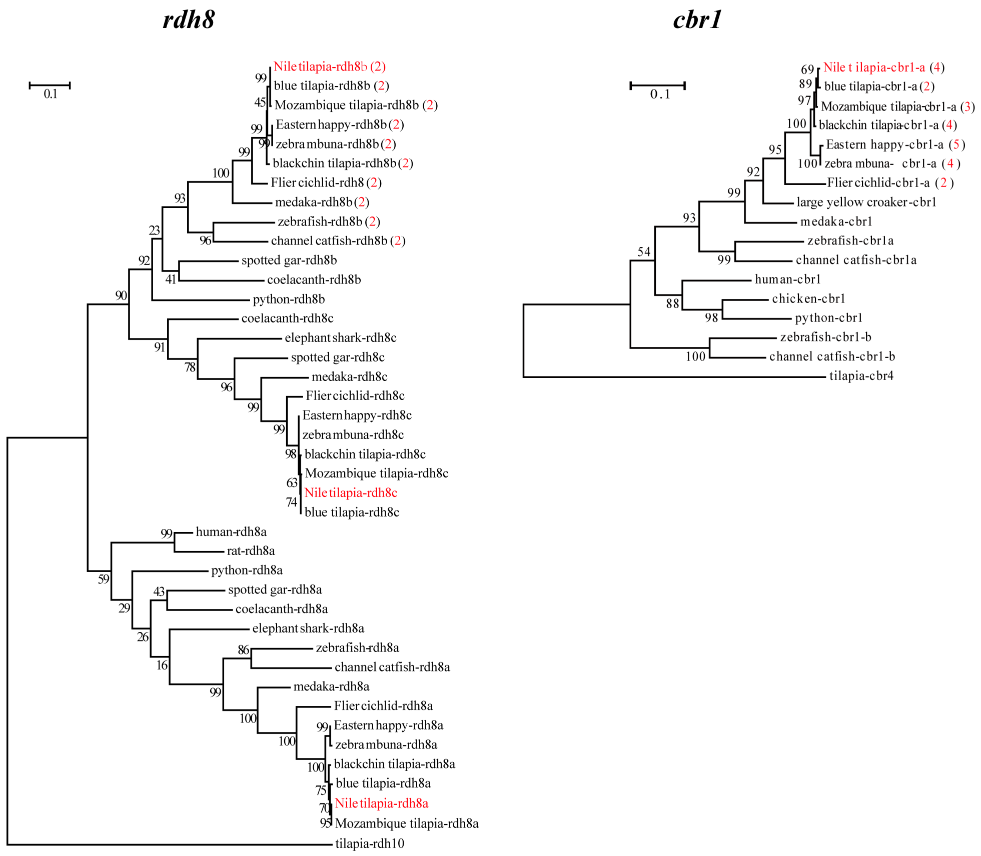

Figure S2 Phylogenetic trees of *cbr1* and *rdh8*. The trees were constructed using their amino acid sequences of different species and ML method. The genes of Nile tilapia were marked with red. GenBank accession numbers of the sequences used are listed in Table S3-S18. Numbers at the branch of the phylogenetic trees stand for bootstrap. Genes were named according to these phylogenetic trees.

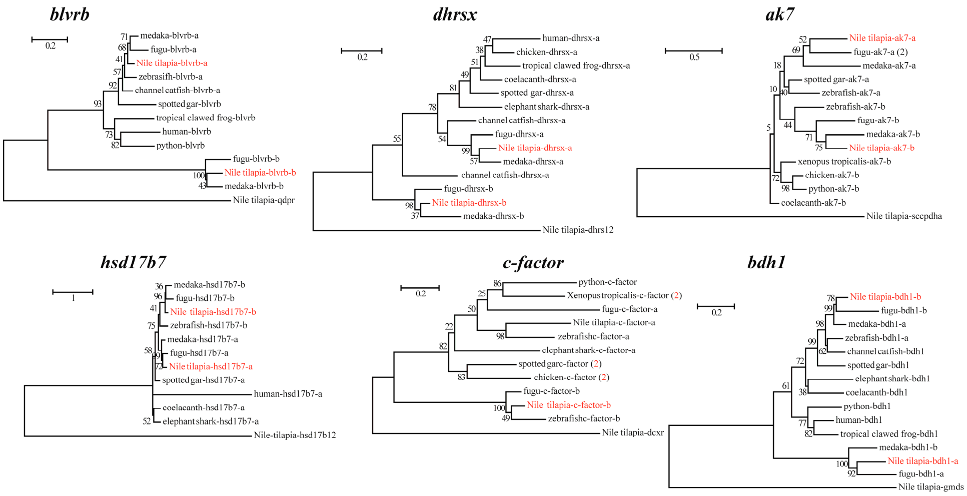

Figure S3 Phylogenetic trees of genes originate from 3R-WGD. The trees were also constructed using their amino acid sequences of different species and ML method. The genes of Nile tilapia were marked with red. GenBank accession numbers of the sequences used are listed in Table S3-S18. Numbers at the branch of the phylogenetic trees stand for bootstrap. Genes were named according to these phylogenetic trees.

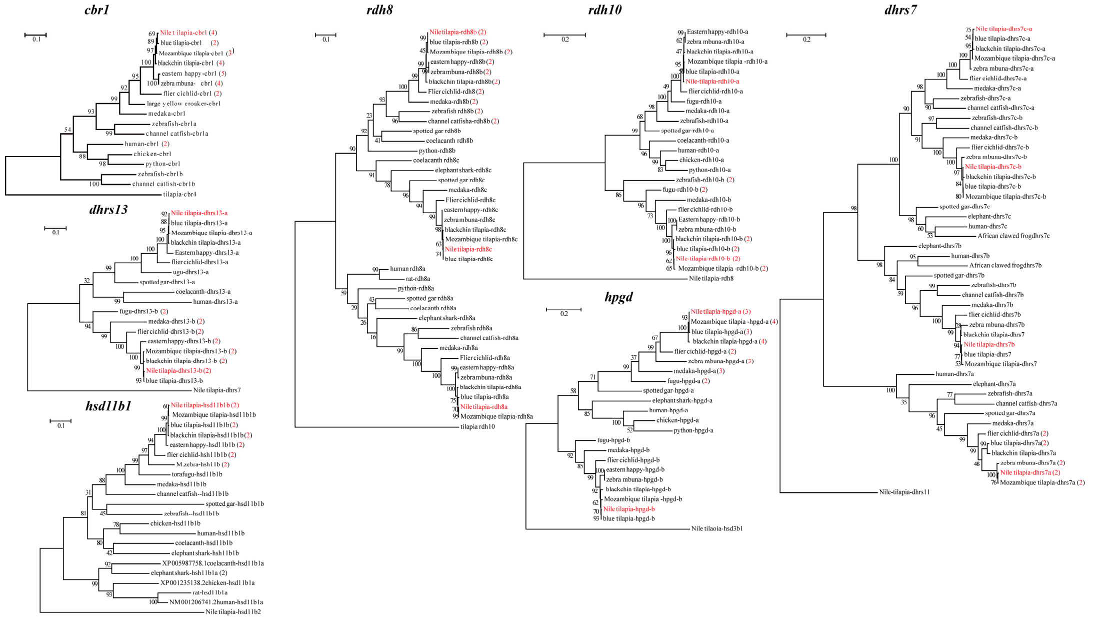

Figure S4 Phylogenetic trees of genes originate from tandem duplication. The trees were also constructed using their amino acid sequences of different species and ML method. The genes of Nile tilapia were marked with red. GenBank accession numbers of the sequences used are listed in Table S3-S18. Numbers at the branch of the phylogenetic trees stand for bootstrap. Genes were named according to these phylogenetic trees.

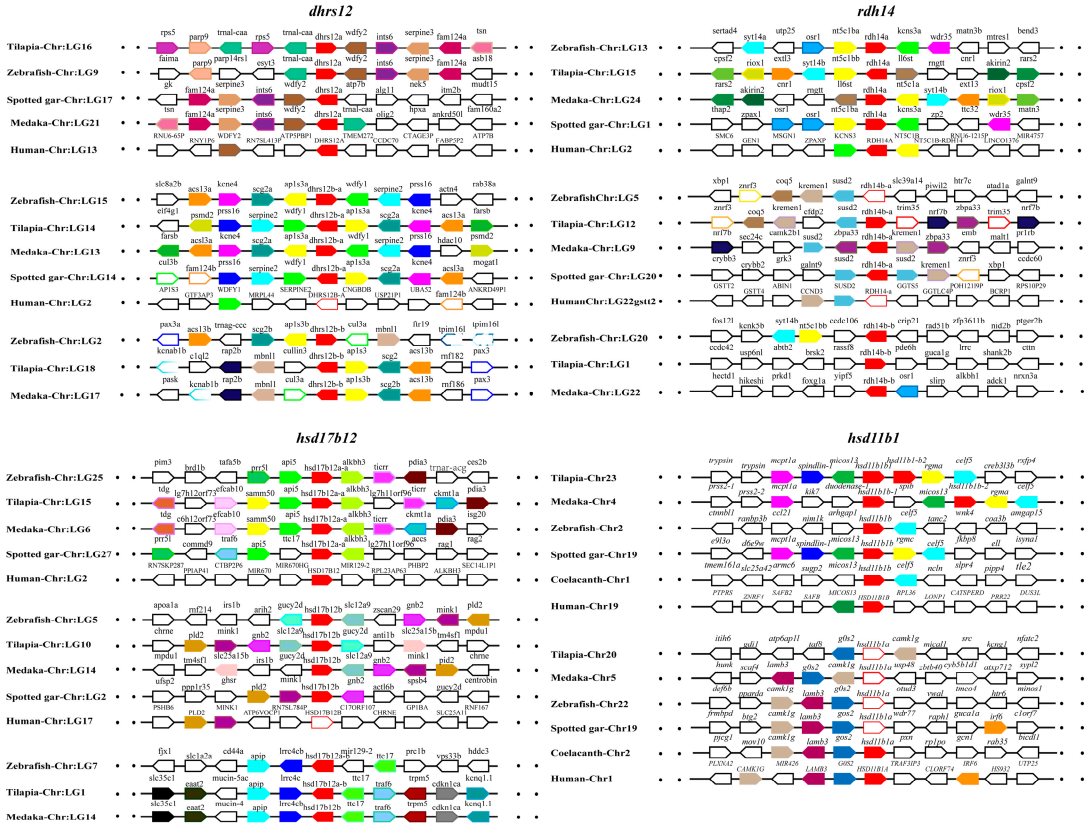

Figure S5 Syntenic analysis of genes originated from 2R-WGD and their adjacent genes in tilapia and other vertebrates. Rectangles represent genes in chromosome/scaffold. Dots represent omitted genes of the chromosome/scaffold. The direction of the arrows indicates the gene orientation. The *SDR*s are shown in red, while the other genes are shown in different colors.

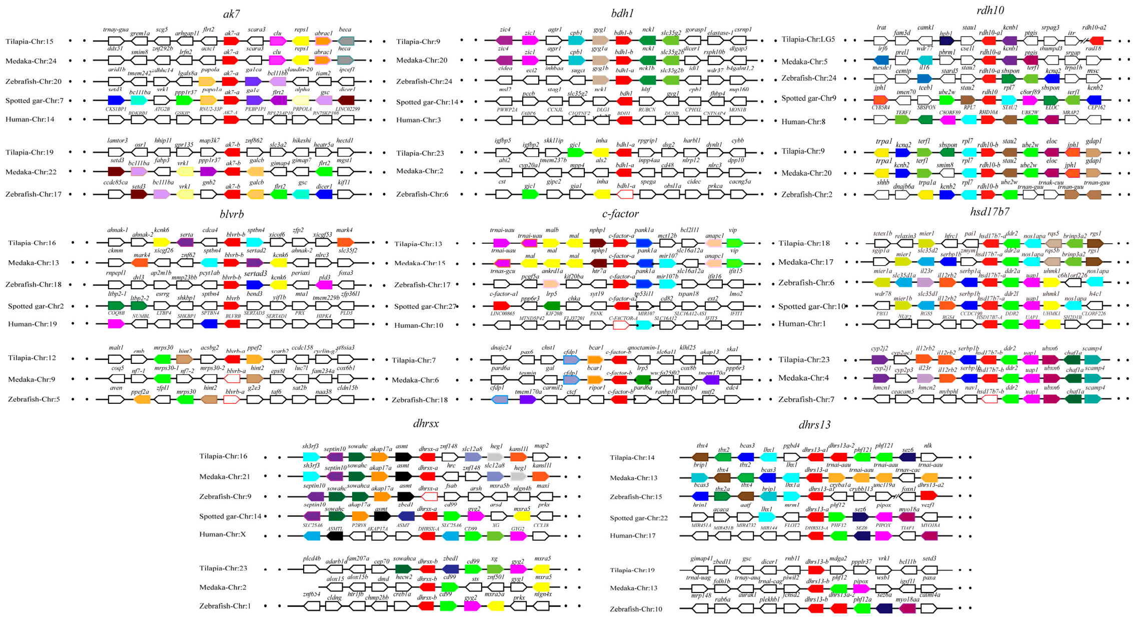

Figure S6 Syntenic analysis of genes originated from 3R-WGD and their adjacent genes in tilapia and other vertebrates. Rectangles represent genes in chromosome/scaffold. Dots represent omitted genes of the chromosome/scaffold. The direction of the arrows indicates the gene orientation. The *SDR*s are shown in red, while the other genes are shown in different colors.

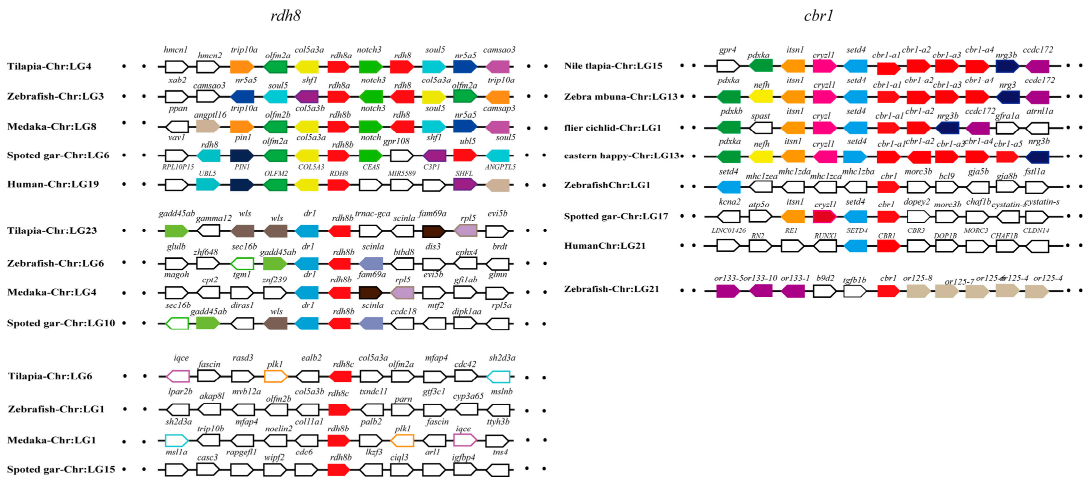

Figure S7 Syntenic analysis of *cbr1* and *rdh8* and their adjacent genes in tilapia and other vertebrates. Rectangles represent genes in chromosome/scaffold. Dots represent omitted genes of the chromosome/scaffold. The direction of the arrows indicates the gene orientation. The *SDR*s are shown in red, while the other genes are shown in different colors.

gene	5dXX	5dXY	30dXX	30dXY	3mXX	3mXY	6mXX	6mXY
decr2	10.7	15.5	22.1	15.0	210.6	13.5	145.2	7.4
dhrs3	5.6	0.2	33.8	64.0	93.8	12.8	114.2	7.1
dhrs7b	9.7	11.9	16.5	13.8	218.3	26.4	165.6	22.7
dhrs9	4.0	2.2	30.1	18.5	93.8	28.8	122.1	14.8
far1	4.0	2.9	8.4	10.1	164.7	13.5	169.2	8.9
hpgd- b1	0.9	2.0	64.6	57.8	271.6	32.9	172.5	6.0
hsd3b7	0.2	0.1	44.2	35.9	412.9	20.7	387.0	25.4
rdh10b - 1	0.0	0.2	2.5	1.7	61.7	0.8	72.3	0.8
rdh12b	20.7	21.3	28.1	18.3	151.2	24.8	143.3	21.0
sdr39u1	2.2	7.2	20.3	17.1	91.1	14.8	107.6	24.9
spra	12.5	16.9	24.0	14.0	167.4	22.5	218.1	12.3
ak7- a	5.8	3.2	1.9	1.1	2.0	97.1	0.7	103.3
hsd3b1	1.8	1.8	379.5	348.2	61.5	1018.4	64.7	475.9
hsd11b1 - b1	0.4	0.0	16.2	12.2	2.4	24.8	1.3	25.2
hsd11b2	32.9	19.1	9.6	10.9	3.3	112.0	15.8	174.5
rdh14a - b	0.1	0.0	7.5	6.5	20.3	124.7	25.7	125.5
kdsr	6.6	4.3	8.6	10.6	28.8	201.0	29.0	193.8

Table S8 The expression profiles (RPKM) of *SDR*s in the tilapia gonads. These genes were selected from gonad transcriptome data based on their high and sexual dimorphic expression. *hsd3b7*, *rdh10b-2 rdh12b*, *dhrs3*, *dhrs7b*, *dhrs9*, *sdr39u1*, *hpgd-b1*, *far1*, *spra* and *decr2* were ovary-enriched genes, while *hsd3b1*, *hsd11b1-b2*, *hsd11b2*, *rdh14a-b*, *kdsr* and *ak7a* were testis-enriched genes.
