# Supplementary material for: Identification, Expression and Evolution of Short-Chain Dehydrogenases/Reductases in Nile Tilapia (Oreochromis niloticus)
Source: Int J Mol Sci. 2021 Apr 18;22(8):4201. doi: 10.3390/ijms22084201 (PMC8073704; doi:10.3390/ijms22084201)
Supplement: Supplementary file 1 [file ijms-22-04201-s001.zip › Revised Supplemental Tables.pdf]

Table S1 Rainbow trout (*Oncorhynchus mykis*) S*DR* gene name, accession number and chromosome location

Gene name	Accession No.	Chr. location	Gene name	Accession No.	Chr. location
ak7	XP_021429573	19	*dhrsx*	XP_021464515	7
ak7	XP_021467117	8	*far1*	XP_021458526	1
bdh1	XP_021430311	19	*far1*	XP_021431195	2
bdh1	XP_021475691	11	*far1*	XP_021419562	15
bdh2	XP_021423991	17	*far1*	XP_021450356	21
blvrb	XP_021461223	6	*far1*	XP_021432753	21
blvrb	XP_021443148	27	*fasn*	XP_021431901	20
blvrb	XP_021437673	24	*fasn*	XP_021436965	23
c-factor	XP_021420311	16	*gale*	NP_001158720	19
c-factor	XP_021445745	1	*gale*	XP_021428813	19
c-factor	XP_021426205	2	*gale*	XP_021440304	25
c-factor	XP_036810540	2	*kdsr*	XP_021469106	8
cbr1	XP_021464592	7	*gmds*	XP_021457106	5
cbr1	XP_021453441	3	*hpgd*	XP_021429149	19
cbr1	NP_001118068	Y	*hpgd*	XP_021475042	10
cbr1	XP_021447008	29	*hpgd*	XP_021420121	2
cbr1	NP_001117727	Y	*hsd3b1*	XP_021453392	3
cbr4	XP_021455030	30	*hsd3b7*	XP_021481251	12
cbr4	XP_021457094	5	*hsd11b1*	XP_021459442	5
dcxr	XP_021431894	20	*hsd11b1*	XP_021454629	30
decr1	XP_021413978	2	*hsd11b2*	XP_036819332	26
decr2	XP_021420863	16	*hsd11b2*	NP_001117690	6
dhrs1	XP_021444695	28	*hsd17b1*	XP_021415127	13
dhrs3	XP_021465438	7	*hsd17b1*	XP_021480528	12
dhrs3	XP_021425156	11	*hsd17b2*	XP_021431966	2
dhrs3	XP_021471419	9	*hsd17b3*	XP_021462106	6
dhrs3	XP_021422168	16	*hsd17b3*	XP_021477690	11
dhrs4	XP_021470613	9	*hsd17b4*	XP_021460921	6
dhrs4	XP_021433555	21	*hsd17b7*	XP_021468474	8
dhrs7cb	XP_021431235	20	*hsd17b8*	XP_021416231	32
dhrs7cb	XP_021414021	13	*hsd17b10*	XP_021422861	16
dhrs7b	XP_021480024	12	*hsd17b12*	XP_021473840	10
dhrs7	XP_021440629	65	*hsd17b12*	XP_021421786	2
dhrs9	XP_021434749	22	*hsd17b12*	XP_021454377	30
dhrs11	XP_021479276	12	*hsd17b14*	XP_021481191	12
dhrs11	XP_021443635	27	*hsd17b14*	XP_021414076	13
dhrs11	XP_021473692	10	*hsdl1*	XP_021456082	4
dhrs12	XP_021453287	3	*hsdl2*	XP_021478132	12
dhrs12	XP_021438605	24	*htatip2*	XP_021462511	6
dhrs12	XP_021444465	28	*htatip2*	XP_021441306	26
dhrs12	XP_021444014	27	*mat2b*	XP_021473230	10
dhrs13	XP_021443939	27	*ndufa9*	XP_021457803	1
dhrs13	XP_021443941	27	*ndufa9*	XP_021430579	2
dhrs13	XP_021474125	10	*nsdhl*	XP_021439009	31
dhrs13	XP_021438536	24	*nsdhl*	XP_021417438	31

Gene name	Accession No.	Chr. location	Gene name	Accession No.	Chr. location
dhrsx	XP_021427092	18	*pecr*	XP_021469836	8
qdpr	XP_021417106	14	*rdh13*	XP_021451495	3
qdpr	XP_021439812	31	*rdh14*	XP_021430545	19
qdpr	XP_021421399	16	*rdh14*	XP_021456669	4
rdh3	NP_001158553	3	*rdhe2*	XP_021418482	15
rdh7	XP_021464353	10	*rdhe2*	XP_021468752	8
rdh7	XP_021426893	18	*rdhe2*	XP_021445344	28
rdh8	XP_021480901	12	*rdhe2*	XP_021467720	8
rdh8	XP_021413594	13	*rdhe2*	XP_021467720	8
rdh8	XP_021425892	Un	*sdr39u1*	XP_021469445	8
rdh8	XP_021460113	5	*sdr42e1*	XP_021442224	26
rdh8	XP_021413596	13	*sdr42e2*	XP_036797394	13
rdh10	XP_021445086	28	*sccpdh*	XP_021440191	25
rdh10	XP_021477965	15	*sccpdh*	XP_021468103	8
rdh10	XP_021419980	11	*sccpdh*	XP_021430351	19
rdh11	XP_021427767	18	*spr*	XP_021446646	Y
rdh11	XP_021476077	11	*tdgs*	XP_021464223	7
rdh11	XP_021474125	6	*tdh*	XP_021454217	4
rdh12	XP_021427129	2	*tdh*	XP_021430224	19
rdh12	XP_021427765	18	*tdh*	XP_021438777	25
rdh12	XP_021416141	32	*tdh*	XP_021466965	8
rdh12	XP_021474385	2	*tgds*	XP_021426701	18
rdh12	XP_021418413	15	*tgds*	XP_021426699	18
rdh12	XP_021476076	11	*tsta3*	XP_021455258	30
rdh12	XP_021466947	6	*tsta3*	XP_021460170	5
rdh12	XP_021441147	25	*tsta3*	XP_021411953	13
rdh12	NP_001153986	12	*uxs1*	XP_021453612	3
rdh12	XP_021430594	14	*uxs1*	XP_021435620	22
rdh13	XP_021420862	16	*wwox*	XP_021428369	2

Table S2 Common carp (*Cyprinus carpio*) *SDR* gene name, accession number and chromosome location

Gene name	Accession No.	Chr. location	Gene name	Accession No.	Chr. location
ak7	XP_018981844	40	*hsd17b12*	XP_018933276	Un
ak7	XP_018965407	18	*hsd17b12*	XP_018960136	10
bdh1	XP_018922274	47	*hsd17b14*	XP_018937015	7
bdh1	XP_018922275	47	*hsdl1*	XP_018933014	Un
bdh2	XP_018970238	26	*hsdl1*	XP_018981981	40
blvrb	XP_018977991	35	*hsdl2*	XP_018935422	Un
cbr1	XP_018965340	2	*hsdl2*	XP_018935408	Un
dcxr	XP_018968200	23	*htatip2*	XP_018937032	Un
decr1	XP_018975475	32	*htatip2*	XP_018955465	Un
decr1	XP_018941224	Un	*htatip2*	XP_018961909	13
decr2	XP_018980732	38	*htatip2*	XP_018937024	Un
decr2	XP_018922675	47	*gmds*	XP_018918317	40
dhrs1	XP_018980227	38	*gmds*	XP_018931205	Un
dhrs1	XP_018935495	Un	*kdsr*	XP_018923561	48
dhrs1	XP_018941239	Un	*mat2b*	XP_018932190	Un
dhrs3	XP_018962800	Un	*ndufa9*	XP_018923755	49
dhrs3	XP_018927859	Un	*ndufa9*	XP_018923769	49
dhrs3	XP_018944462	Un	*nsdhl*	XP_018971472	28
dhrs3	XP_018943908	Un	*nsdhl*	XP_018931863	Un
dhrs4	XP_018962194	13	*qdpr*	XP_018946443	Un
dhrs4	XP_018937241	Un	*qdpr*	XP_018927669	Un
dhrs4	XP_018928537	Un	*qdpr*	XP_018970266	26
dhrs7	XP_018929771	6	*rdh7*	XP_018918774	41
dhrs7	XP_018934049	Un	*rdh7*	XP_018936082	6
dhrs7	XP_018973425	31	*rdh7*	XP_018935554	6
dhrs7	XP_018933616	Un	*rdh8*	XP_018927573	Un
dhrs7	XP_018934402	Un	*rdh8*	XP_018961309	12
dhrs11	XP_018939666	Un	*rdh8*	XP_018966742	20
dhrs11	XP_018939023	Un	*rdh8*	XP_018958430	Un
dhrs11	XP_018938966	Un	*rdh8*	XP_018954556	Un
dhrs12	XP_018935156	Un	*rdh10*	XP_018922594	47
dhrs12	XP_018949536	9	*rdh10*	XP_018969988	3
dhrs12	XP_018968787	3	*rdh10*	XP_018949821	Un
dhrs12	XP_018951236	Un	*rdh11*	XP_018923485	33
dhrs12	XP_018964942	17	*rdh11*	XP_018923485	48
dhrs12	XP_018954091	Un	*rdh12*	XP_018918380	40
dhrs12	XP_018918508	40	*rdh12*	XP_018948397	Un
dhrs12	XP_018918603	41	*rdh12*	XP_018923484	48
dhrs13	XP_018951324	Un	*rdh12*	XP_018948393	Un
dhrsx	XP_018963643	2	*rdh12*	XP_018918603	40
far1	XP_018977831	35	*rdh12*	XP_018918508	40
far1	XP_018926033	Un	*rdh14*	XP_018927025	Un
far1	XP_018944148	8	*rdh16*	XP_018944710	Un
far1	XP_018977833	35	*sccpdh*	XP_018974229	31
fasn	XP_018943534	Un	*sccpdh*	XP_018935265	Un
fasn	XP_018926202	Un	*sccpdh*	XP_018971394	3

Gene name	Accession No.	Chr. location	Gene name	Accession No.	Chr. location
hpgd	XP_018949277	Un	*sccpdh*	XP_018946978	Un
hpgd	XP_018956596	Un	*sdr39u1*	XP_018936128	Un
hsd3b1	XP_018925427	Un	*sdr42e2*	XP_018961426	12
hsd3b7	XP_018934915	6	*sdr42e2*	XP_018950261	Un
hsd3b7	XP_018927542	6	*sdr42e2*	XP_018947610	Un
hsd11b1	XP_018963376	14	*spr*	XP_018926689	Un
hsd11b1	XP_018921668	45	*spr*	XP_018964036	16
hsd11b2	XP_018924670	Un	*spr*	XP_018920157	43
hsd11b2	XP_018926965	Un	*tdh*	XP_018941393	Un
hsd11b2	XP_018932885	6	*tdh*	XP_018977106	34
hsd17b1	XP_018954756	Un	*tdh2*	XP_018977104	34
hsd17b2	XP_018970527	27	*tgds*	XP_018932716	Un
hsd17b2	XP_018970521	27	*tgds*	XP_018944364	Un
hsd17b3	XP_018971370	3	*tsta3*	XP_018981880	10
hsd17b4	XP_018970268	26	*tsta3*	XP_018981895	40
hsd17b7	XP_018956596	11	*tsta3*	XP_018981883	40
hsd17b8	XP_018979734	37	*tsta3*	XP_018981892	40
hsd17b12	XP_018953731	9	*tsta3*	XP_018943596	Un
hsd17b12	XP_018937767	1	*usx1*	XP_018964921	17
hsd17b12	XP_018936083	Un	*usx1*	XP_018979215	36
hsd17b12	XP_018920176	43	*wwox*	XP_018922850	48

Table S3 Tilapia (*Oreochromis niloticus*) *SDR* gene name, accession number and chromosome location

Gene name	Accession No.	Chr. location	Gene name	Accession No.	Chr. location
ak7-a	XP_005475123	LG15	*hsd11b1l-b1*	XP_003451366	LG23
ak7-b	XP_013121720	LG19	*hsd11b1l-b2*	XP_003451366	LG23
bdh1-a	XP_003439253	LG9	*hsd11b2*	NP_001266686	LG1
bdh1-b	XP_003453238	LG23	*hsd17b1*	NP_001266724	LG4
bdh2	XP_005458791	LG6	*hsd17b3*	XP_019221518	LG12
blvrb-a	XP_005459830	LG12	*hsd17b4*	XP_003451110	LG12
blvrb-b	XP_003456428	LG14	*hsd17b7-b*	XP_003453353	LG18
c-factor-a	XP_003438269	LG13	*hsd17b7-a*	XP_003439749	LG18
c-factor-b	XP_00545429	LG7	*hsd17b8*	NP_001266465	LG22
cbr1-a1	XP_003438400	LG13	*hsd17b10*	XP_003457109	LG20
cbr1-a2	XP_025753474	LG13	*hsd17b12a-a*	NP_001266727	LG10
cbr1-a3	NP_001266577	LG13	*hsd17b12b*	XP_003450847	LG1
cbr1-a4	XP_003438402	LG13	*hsd17b12a-b*	XP_003455445	LG7
cbr4	XP_003439992	LG23	*hsd17b14*	XP_003442566	LG4
dcxr	XP_003454090	LG8	*hsdl1*	XP_003456679	LG15
decr1	XP_003457613	LG11	*hsdl2*	XP_003440257	LG7
decr2	XP_003438502	LG8	*htatip2*	XP_003454377	LG1
dhrs1	XP_003457459	LG18	*kdsr*	XP_003438177	LG18
dhrs3	XP_003447727	LG5	*ndufa9*	XP_003447104	LG7
dhrs4	XP_013122331	LG3	*nsdhl*	XP_003445987	LG2
dhrs7b	XP_003454026	LG94	*mat2b*	XP_003457059	LG10
dhrs7c-a	XP_003438705	LG8	*pecr*	XP_019203563	LG18
dhrs7c-b	XP_005469101	LG8	*qdpra*	XP_003443461	LG2
dhrs7a-2	XP_019204754	LG19	*rdhe2*	XP_005461579	LG18
dhrs7a-1	XP_003453138	LG19	*rdh3*	XP_003440906	LG23
dhrs9	XP_003447377	LG16	*rdh5*	XP_003456138	LG5
dhrs11-a1	XP_003440183	LG14	*rdh7*	XP_013128137	LG16
dhrs11-a2	XP_003440325	LG14	*rdh8c*	XP_003444247	LG23
dhrs11-a3	XP_003440326	LG14	*rdh8b-2*	XP_003442138	LG4
dhrs11-a4	XP_003440184	LG14	*rdh8a*	XP_003457799	LG6
dhrs11-a5	XP_025754019	LG14	*rdh8b-1*	XP_003442264	LG4
dhrs11-a6	XP_019223060	LG14	*rdh10-a1*	XP_013128689	LG5
dhrs11-a7	XP_019223058	LG14	*rdh10a-b*	XP_003443518	LG9
dhrs11-a8	XP_019223059	LG14	*rdh10-a2*	XP_013124696	LG5
dhrs11-a9	XP_003440187	LG14	*rdh11-b*	XP_003440457	LG7
dhrs11-a10	XP_019223051	LG14	*rdh11-a*	XP_003442756	LG5
dhrs11-a11	XP_019223053	LG14	*rdh12b*	XP_003439324	LG9
dhrs11-a12	XP_013123946	LG14	*rdh12a*	XP_005462441	LG11
dhrs11-a13	XP_003440189	LG14	*rdh12c-a*	XP_003445520	LG19
dhrs11-b	XP_003456202	LG10	*rdh12c-b*	XP_003443556	LG9
dhrs12a	XP_003454954	LG16	*rdh13-a*	XP_003437667	LG1
dhrs12b-a	XP_005476304	LG16	*rdh13-b*	XP_003438653	LG8
dhrs12b-b	XP_003456991	LG16	*rdh14b-b*	XP_003446328	LG15
dhrs13-a2c	XP_003459274	LG19	*rdh14b-a*	XP_003454190	LG12
dhrs13-a1	XP_003459273	LG19	*rdh14a*	XP_003452656	LG1
dhrs13-b	XP_003458484	LG19	*sccpdha*	XP_003454570	LG1

Gene name	Accession No.	Chr.location	Gene name	Accession No	Chr.location
dhrsx-a	XP_005463734	LG23	*sdr39u1*	XP_003447514	LG18
dhrsx-b	XP_025759728	LG16	*sdr42e1*	XP_013120237	LG4
far1	XP_003452079	LG17	*sdr42e2*	XP_013120237	LG4
far2	XP_019216357	LG7	*spra*	XP_003453684	LG7
fasn	XP_003454104	LG8	*syt12*	XP_005449457	LG7
gale	XP_013127364	LG19	*tdh1*	XP_025758862	LG23
gmds	XP_003457343	LG17	*tdh2*	XP_003455669	LG15
hpgd-a1	XP_003451464	LG10	*tdh3*	XP_003446277	LG19
hpgd-a2	XP_003451465	LG10	*tgds*	XP_013119972	LG23
hpgd-a3	XP_003451496	LG10	*tsta3*	XP_003453582	LG17
hpgd-b	XP_003456541	LG6	*uxs1*	XP_003452998	LG16
hsd3b1	NP_001266438	LG16	*vcr*	XP_003454090	LG8
hsd3b7	NP_001266602	LG4	*wwox*	XP_013124094	LG7

Table S4 Zebra mbuna (*Maylandia zebra*) *SDR* gene name, accession number and chromosome location

Gene name	Accession No.	Chr. location	Gene name	Accession No.	Chr. location
ak7	XP_004550614	15	*hsd17b1*	XP_004552340	4
ak7	XP_024657691	Un	*hsd17b3*	XP_004547253	12
bdh1	XP_004546828	9	*hsd17b4*	XP_014269940	12
bdh2	XP_004549579	6	*hsd17b7*	XP_004554911	23
blvrb	XP_004569667	14	*hsd17b7*	XP_004567183	18
blvrb	XP_004575692	12	*hsd17b8*	XP_014269251	22
bdh1	XP_004562605	23	*hsd17b10*	XP_014262903	20
c-factor	XP_004553475	Un	*hsd17b12*	XP_014264573	7
c-factor	XP_004564655	7	*hsd17b12*	XP_004558130	1
c-factor	XP_004556304	7	*hsd17b12*	XP_004564134	10
cbr1	XP_004553608	13	*hsd17b14*	XP_004552560	Un
cbr1	XP_014263238	13	*hsdl1*	XP_004550709	15
cbr1	XP_004553602	13	*hsdl2*	XP_004561932	7
cbr1	XP_004553601	13	*htatip2*	XP_004571108	1
cbr4	XP_004557152	23	*kdsr*	XP_004542772	18
dcxr	XP_014265540	Un	*mat2b*	XP_024659608	10
decr1	XP_024656267	Un	*ndufa9*	XP_012779317	7
decr2	XP_004562538	Un	*nsdhl*	XP_004541051	2
dhrs1	XP_012778475	18	*pecr1*	XP_014265063	18
dhrs3	XP_004547505	5	*qdpra*	XP_004553710	2
dhrs4	XP_004575327	3	*rdh1*	XP_004557740	16
dhrs7	XP_004575993	8	*rdhe2*	XP_014266734	18
dhrs7	XP_024657947	4	*rdh5*	XP_023010851	Un
dhrs7	XP_004556418	4	*rdh7*	XP_023009772	23
dhrs7	XP_004540620	19	*rdh8*	XP_004552958	23
dhrs7	XP_004540622	19	*rdh8*	XP_004552166	4
dhrs9	XP_004557741	16	*rdh8*	XP_004552168	4
dhrs11	XP_004557490	10	*rdh8*	XP_004569064	6
dhrs11	XP_024660739	14	*rdh10*	XP_004546674	10
dhrs11	XP_024660820	14	*rdh10*	XP_004544880	5
dhrs11	XP_024660738	14	*rdh11*	XP_004546591	Un
dhrs11	XP_014262724	14	*rdh12*	XP_004540029	19
dhrs11	XP_023007967	14	*rdh12*	XP_004551959	9
dhrs11	XP_024660822	14	*rdh12*	XP_024660086	11
dhrs11	XP_024660740	14	*rdh12*	XP_004546592	Un
dhrs11	XP_014264343	14	*rdh12*	XP_014266574	Un
dhrs12	XP_004569008	16	*rdh12*	XP_024658194	5
dhrs12	XP_004542660	Un	*rdh13*	XP_004562537	Un
dhrs12	XP_014264996	14	*rdh13*	XP_004553247	7
dhrs13	XP_004572239	Un	*rdh13*	XP_004539646	1
hsd11b1	XP_004552835	23	*dhrs13*	XP_004572843	Un
hsd11b2	XP_004539907	1	*dhrs13*	XP_004572844	Un
dhrsx	XP_014263754	16	*rdh14*	XP_004571894	1
dhrsx	XP_004564181	23	*rdh14*	XP_004550533	15
far1	XP_004566142	7	*rdh14*	XP_004566725	Un
far1	XP_004548437	17	*rdh16*	XP_030603101	2

Gene name	Accession No.	Chr. location	Gene name	Accession No.	Chr. location
fasn	XP_014263922	Un	*sccpdh*	XP_004539560	1
gale	XP_004566599	19	*sdr39u1*	XP_004555254	18
gmds	XP_004571820	17	*sdr42e1*	XP_004539763	1
hpgd	XP_004538724	6	*sdr42e2*	XP_004558551	4
hpgd	XP_004561840	10	*spra*	XP_012774836	6
hpgd	XP_014264743	10	*syt12*	XP_030587397	6
hpgd	XP_004561841	10	*tgds*	XP_024654445	23
hsd3b1	XP_004573053	16	*tsta3*	XP_014268467	17
hsd3b7	XP_024658013	4	*uxs1*	XP_004551345	16
hsd3b7	XP_004552503	4	*vcr*	XP_030593142	9
hsd11b1	XP_004552836	23	*wwox*	XP_004553403	7

Table S5 Flier cichlid (*Archocentrus centrarchus*) *SDR* gene name, accession number and chromosome location

Gene name	Accession No.	Chr. location	Gene name	Accession No.	Chr. location
ak7	XP_030574781	22	*hsd17b2*	XP_030586797	6
ak7	XP_030577485	24	*hsd17b3*	XP_030593997	9
bdh1	XP_030604021	2	*hsd17b4*	XP_030590823	8
bdh1	XP_030612994	20	*hsd17b4*	XP_030593144	9
bdh2	XP_030612417	1	*hsd17b7*	XP_030582602	4
blvrb	XP_030593902	9	*hsd17b8*	XP_030597318	11
blvrb	XP_030599346	13	*hsd17b10*	XP_030590622	7
c-factor	XP_030604327	15	*hsd17b12*	XP_030588231	6
c-factor	XP_030588385	6	*hsd17b12*	XP_030602164	14
cbr1	XP_030603886	15	*hsd17b12*	XP_030580375	3
cbr1	XP_030603619	15	*hsdl1*	XP_030577380	24
cbr4	XP_030583759	4	*hsdl2*	XP_030598559	12
dcxr	XP_030610727	19	*htatip2*	XP_030581846	3
decr1	XP_030605614	16	*kdsr*	XP_030608201	17
decr2	XP_030611302	19	*mat2b*	XP_030602876	14
dhrs1	XP_030607612	17	*ndufa9*	XP_030587212	6
dhrs3	XP_030584367	5	*nsdhl*	XP_030594935	10
dhrs4	XP_030609163	18	*pecr1*	XP_030608213	17
dhrs7	XP_030574382	22	*qdpra*	XP_030595820	10
dhrs7	XP_030599425	1	*rdh1*	XP_030613615	21
dhrs7	XP_030591237	8	*rdhe2*	XP_030581239	Un
dhrs7	XP_030591988	8	*rdh5*	XP_030585185	5
dhrs7	XP_030611307	19	*rdh7*	XP_030603119	2
dhrs9	XP_030613616	21	*rdh7*	XP_030603080	2
dhrs11	XP_030601935	14	*rdh8*	XP_030591624	8
dhrs11	XP_030601982	14	*rdh8*	XP_030591105	8
dhrs11	XP_030600722	13	*rdh8*	XP_030583917	4
dhrs11	XP_030599679	13	*rdh8*	XP_030586349	1
dhrs11	XP_030599682	13	*rdh10*	XP_030611941	20
dhrs11	XP_030599681	13	*rdh10*	XP_030585020	5
dhrs11	XP_030599678	13	*rdh11*	XP_030585770	5
dhrs11	XP_030600225	13	*rdh12*	XP_030575231	22
dhrs11	XP_030600031	13	*rdh12*	XP_030605626	16
dhrs12	XP_030614159	21	*rdh12*	XP_030612878	20
dhrs12	XP_030599637	13	*rdh12*	XP_030612137	20
dhrs12	XP_030608001	17	*rdh12*	XP_030612136	20
dhrs13	XP_030600692	13	*rdh13*	XP_030610356	19
dhrs13	XP_030574295	22	*rdh13*	XP_030605627	16
dhrs13	XP_030574296	22	*rdh13*	XP_030581826	3
dhrsx	XP_030610108	2	*rdh14*	XP_030577368	24
dhrsx	XP_030613911	21	*rdh14*	XP_030592831	9
far1	XP_030575806	23	*rdh14*	XP_030580401	Un
far1	XP_030586730	6	*rdh16*	XP_030602654	2
fasn	XP_030610501	19	*sccpdh*	XP_030614853	3
gale	XP_030574915	22	*sdr39u1*	XP_030608160	17

Gene name	Accession No.	Chr. location	Gene name	Accession No.	Chr. location
gmds	XP_030583496	4	*sdr42e1*	XP_030582206	3
hpgd	XP_030591291	1	*sdr42e2*	XP_030591555	8
hpgd	XP_030603198	14	*syt12*	XP_030587397	6
hpgd	XP_030603196	14	*tdh*	XP_030600346	2
hsd3b1	XP_030614373	21	*tdh*	XP_030577494	24
hsd3b1	XP_030614413	21	*tdh*	XP_030575268	22
hsd3b7	XP_030592014	8	*tgds*	XP_030605735	2
hsd11b1	XP_030583795	4	*tsta3*	XP_030582535	4
hsd11b1	XP_030582397	4	*uxs1*	XP_030614365	21
hsd11b2	XP_030574951	3	*vcr*	XP_030593142	9
hsd17b1	XP_030592203	8	*wwox*	XP_030587349	6

Table S6 Eastern happy (*Astatotilapia calliptera*) *SDR* gene name, accession number and chromosome location

Gene name	Accession No.	Chr. location	Gene name	Accession No.	Chr. location
ak7	XP_026008552	19	*hsd11b1*	XP_026014800	23
ak7	XP_025998132	15	*hsd11b2*	XP_026031809	1
bdh1	XP_026035443	9	*hsd17b1*	XP_026019446	4
bdh1	XP_026015793	15	*hsd17b3*	XP_026042322	12
bdh2	XP_026026889	6	*hsd17b4*	XP_026042747	12
blvrb	XP_030593902	9	*hsd17b7*	XP_026005584	18
blvrb	XP_026047736	14	*hsd17b7*	XP_026014625	23
c-factor	XP_026045742	13	*hsd17b8*	XP_026012944	22
c-factor	XP_026028755	7	*hsd17b10*	XP_026010592	20
c-factor	XP_026030588	7	*hsd17b12*	XP_026030926	7
cbr1	XP_026045104	13	*hsd17b12*	XP_026016831	1
cbr1	XP_026045105	13	*hsd17b12*	XP_026037968	10
cbr1	XP_026046226	13	*hsd17b14*	XP_026019901	4
cbr1	XP_026046227	13	*hsdl1*	XP_026050713	15
cbr1	XP_026045101	13	*hsdl2*	XP_026030416	7
cbr4	XP_026015766	23	*htatip2*	XP_026033592	1
dcxr	XP_026033310	8	*kdsr*	XP_026004696	18
decr1	XP_026041478	11	*mat2b*	XP_026037310	10
decr2	XP_026034575	8	*ndufa9*	XP_026032475	7
dhrs1	XP_026005297	18	*nsdhl*	XP_026046424	2
dhrs3	XP_026022932	5	*qdpra*	XP_026038788	2
dhrs4	XP_026018030	3	*pecr1*	XP_026005295	18
dhrs7	XP_026019830	4	*rdh1*	XP_025999571	16
dhrs7	XP_026033183	8	*rdhe2*	XP_026006172	18
dhrs7	XP_026021187	4	*rdh5*	XP_026022127	5
dhrs7	XP_026007600	19	*rdh7*	XP_026014457	23
dhrs7	XP_026007602	19	*rdh8*	XP_026014085	23
dhrs9	XP_025999572	16	*rdh8*	XP_026025133	6
dhrs11	XP_026037798	10	*rdh8*	XP_026020410	4
dhrs11	XP_026047127	14	*rdh8*	XP_026020465	4
dhrs11	XP_026047124	14	*rdh10*	XP_026035248	9
dhrs11	XP_026047126	14	*rdh10*	XP_026023742	5
dhrs11	XP_026047125	14	*rdh11*	XP_026024332	5
dhrs11	XP_026047121	14	*rdh11*	XP_026035490	9
dhrs11	XP_026047120	14	*rdh12*	XP_026008430	19
dhrs11	XP_026047123	14	*rdh12*	XP_026035491	9
dhrs11	XP_026047122	14	*rdh12*	XP_026035954	9
dhrs11	XP_026048356	14	*rdh12*	XP_026039579	11
dhrs11	XP_026048337	14	*rdh13*	XP_026034577	8
dhrs12	XP_025999808	16	*rdh13*	XP_026031756	7
dhrs12	XP_026048293	14	*rdh13*	XP_026028066	1
dhrs12	XP_026005370	18	*rdh14*	XP_026041932	12
dhrs12	XP_026005370	18	*rdh14*	XP_026041932	12
dhrs13	XP_026049162	14	*hsd11b1*	XP_026015946	23

Gene name	Accession No.	Chr. location	Gene name	Accession No.	Chr. location
dhrs13	XP_026049388	14	*rdh14*	XP_026050206	15
dhrs13	XP_026007405	19	*rdh14*	XP_025998425	1
dhrsx	XP_026014025	23	*sccpdh*	XP_026026893	1
dhrsx	XP_026000436	16	*sdr39u1*	XP_026005823	18
far1	XP_026028440	7	*sdr42e1*	XP_026029770	1
far1	XP_026003969	17	*sdr42e2*	XP_026020804	4
fasn	XP_026033248	8	*spra*	XP_026030748	7
gale	XP_026007179	19	*tdh*	XP_026050229	15
gmds	XP_026003331	17	*tdh*	XP_026015897	23
hpgd	XP_026025381	6	*tgds*	XP_026013383	23
hpgd	XP_026037702	10	*tsta3*	XP_026004166	17
hpgd	XP_026037701	10	*uxs1*	XP_025999963	16
hsd3b2	XP_026000417	16	*vcr*	XP_026044248	12
hsd3b7	XP_026019982	4	*wwox*	XP_026031977	7
hsd11b1	XP_026015946	23			

Table S7 Large yellow croaker (*Larimichthys crocea*) *SDR* gene name, accession number and chromosome location

Gene name	Accession No.	Chr. location	Gene name	Accession No.	Chr. location
ak7	XP_027134488	V	*hsd17b12*	XP_027137350	VIII
ak7	XP_010745117	XI	*hsd17b14*	XP_010744565	XII
bdh1	XP_019114125	XXIII	*hsdl1*	XP_010747726	XI
bdh1	XP_019126683	XIX	*hsdl2*	XP_027137969	IX
bdh2	XP_019112525	X	*htatip2*	XP_019123588	VIII
blvrb	XP_027136561	VII	*kdsr*	XP_010728300	II
blvrb	XP_019111216	III	*nsdhl*	XP_019128067	I
cbr1	XP_027133720	III	*ndufa9*	XP_019131679	XXI
cbr4	XP_019133819	XVII	*mat2b*	XP_027129704	XXII
dcxr	XP_019126803	XVI	*pecr*	XP_010754026	X
decr1	XP_010751813	Un	*qdpr*	XP_010729713	VII
decr2	XP_010730054	XVI	*rdhe2*	XP_027132337	Un
dhrs1	XP_019112774	X	*rdh5*	XP_027135010	VI
dhrs3	XP_010727826	VI	*rdh7*	XP_010738548	XIX
dhrs4	XP_010746539	:I	*rdh7*	XP_019110810	XVIII
dhrs7c	XP_010743398	XII	*rdh8*	XP_019114655	XII
dhrs7b	XP_019113805	XII	*rdh8*	XP_010728659	XII
dhrs7	XP_010739765	XVI	*rdh8*	XP_027138691	X
dhrs7	XP_010732469	XXIV	*rdh10*	XP_019118260	VI
dhrs11	XP_010734835	XXII	*rdh10*	XP_027130111	XXIII
dhrs11	XP_010737979	VII	*rdh10*	XP_010740665	VI
dhrs12	XP_019126363	XVII	*rdh11*	XP_027134560	V
dhrs12	XP_010748305	VII	*rdh11*	XP_027146388	XVII
dhrs12	XP_019117711	XII	*rdh11*	XP_010727690	XXI
dhrs13	XP_010749478	VII	*rdh11*	XP_010741587	VI
dhrs13	XP_010736583	V	*rdh12*	XP_010749731	XXIV
dhrs13	XP_019118748	V	*rdh12*	XP_019131806	XXIII
dhrsx	XP_019123632	XVIII	*rdh12*	XP_019110511	XXIII
dhrsx	XP_027137108	VIII	*rdh12*	XP_010733755	XXIII
far1	XP_019134006	XX	*rdh12*	XP_027134542	V
far1	XP_027127930	XX	*rdh13*	XP_010730056	XVI
fasn	XP_027144906	XVI	*rdh13*	XP_027131135	Un
gale	XP_010729589	XXIV	*rdh13*	XP_010754832	VIII
gmds	XP_027145847	XVII	*rdh13*	XP_027128453	XXI
hpgd	XP_010727660	X	*rdh14*	XP_019118821	XI
hpgd	XP_027129665	X	*rdh14*	XP_010731275	III
hpgd	XP_027129533	X	*rdh14*	XP_010733722	V
hsd3b1	XP_010732334	XVIII	*sccpdh*	XP_010730613	VIII
hsd3b7	XP_010743256	XII	*sdr39u1*	XP_010752236	II
hsd11b1	XP_010731274	XVII	*sdr42e1*	XP_019129845	VIII
hsd11b2	XP_010754262	VIII	*sdr42e2*	XP_027141478	XII
hsd17b1	XP_010737963	:XII	*spr*	XP_010752834	IV
hsd17b3	XP_010731296	III	*syt12*	XP_019132207	XVIII

Gene name	Accession No.	Chr. location	Gene name	Accession No.	Chr. location
hsd17b4	XP_010731495	III	*tdh*	XP_019111116	:XIX
hsd17b7	XP_027146397	XVII	*tdh*	XP_010733568	:XI
hsd17b7	XP_019134783	XII	*tdh*	XP_019116859	:XXIV
hsd17b8	XP_027142483	XIII	*tgds*	XP_027131858	Un
hsd17b10	XP_010740929	XV	*tsta3*	XP_027146432	XVII
hsd17b12	XP_027143858	XIV	*uxs1*	XP_027146974	XVIII
hsd17b12	XP_010751836	XXII	*wwox*	XP_027128572	XXI
hsd17b12	XP_019125007	XXII			

Table S8 Medaka (*Oryzias latipes*) *SDR* gene name, accession number and chromosome location

Gene name	Accession No.	Chr. location	Gene name	Accession No.	Chr. location
ak7	XP_011488848	22	*hsd17b10*	XP_004071072	7
ak7	XP_020570347	24	*hsd17b12*	XP_004076567	14
bdh1	XP_023817415	2	*hsd17b12*	XP_004069451	6
bdh1	XP_004081138	20	*hsd17b12*	XP_004066896	3
bdh2	XP_023812785	1	*hsd17b14*	XP_004071213	8
blvrb	XP_004072091	9	*hsdl1*	XP_004083931	24
blvrb	XP_004075298	13	*hsdl2*	XP_023816475	12
c-factor	XP_004077326	15	*htatip2*	XP_004066870	3
c-factor	XP_023811291	6	*kdsr*	XP_004079405	17
cbr1	XP_023818701	15	*nsdhl*	XP_004073407	10
cbr4	XP_004067613	4	*ndufa9*	XP_020559925	6
dcxr	XP_023805429	19	*mat2b*	XP_004076715	14
decr1	XP_004086742	16	*pecr*	XP_004079064	17
decr2	XP_004080392	19	*qdpra*	XP_004086516	10
dhrs1	XP_023820538	17	*rdhe2*	XP_023820697	17
dhrs3	XP_004069360	5	*rdh5*	XP_004068760	5
dhrs4	NP_001264169	18	*rdh7*	XP_004066712	2
dhrs7c	XP_004080752	19	*rdh8*	XP_004071663	8
dhrs7b	XP_004071568	8	*rdh8*	XP_004071404	8
dhrs7	XP_004082333	22	*rdh8*	XP_004068197	4
dhrs11	XP_020564699	14	*rdh8*	XP_004066030	1
dhrs11	XP_004075364	13	*rdh10*	XP_020568309	20
dhrs12	XP_004081481	21	*rdh10*	XP_020558672	5
dhrs12	XP_011480963	12	*rdh11*	XP_023810779	5
dhrs12	XP_020566721	17	*rdh12*	XP_011488374	22
dhrs13	XP_004075914	13	*rdh12*	XP_023805831	20
dhrs13	XP_004075984	13	*rdh12*	XP_023805832	20
dhrsx	XP_004086057	2	*rdh12*	XP_004081219	20
dhrsx	XP_023806351	21	*rdh12*	XP_020557538	16
far1	XP_023811184	6	*rdh13*	XP_004080572	19
far1	XP_023808016	23	*rdh13*	XP_004069702	6
fasn	XP_004080750	19	*rdh13*	XP_020557541	16
gale	?NP_001158720	22	*rdh14*	XP_004082470	22
gmds	XP_023810155	4	*rdh14*	XP_004083466	24
hpgd	XP_023809607	1	*rdh14*	XP_004072089	9
hpgd	XP_023818315	14	*sccpdh*	XP_004067310	3
hpgd	XP_023818316	14	*sdr39u1*	XP_004078633	17
hsd3b7	XP_023813133	8	*sdr42e1*	XP_004067545	3
hsd3b1	NP_001131037	21	*sdr42e2*	XP_020560976	8
hsd11b1	XP_011471570	4	*spr*	XP_020562483	12
hsd11b1	NP_001098261	4	*syt12*	XP_026031025	7
hsd11b2	XP_004066855	3	*tdh*	XP_020569504	22
hsd11b2	XP_004066855	3	*tdh*	XP_020569504	22
hsd17b1	XP_004071345	8	*tdh*	XP_004083734	24
hsd17b3	XP_004072273	9	*tgds*	XP_023805046	2

Gene name	Accession No.	Chr. location	Gene name	Accession No.	Chr. location
hsd17b4	XP_023814070	9	*tsta3*	XP_004068165	4
hsd17b7	XP_004079131	17	*uxs1*	XP_023806318	21
hsd17b7	XP_004068242	4	*wwox*	XP_004070113	6
hsd17b8	XP_004074105	8			

Table S9 Fugu (*Takifugu rubripes*) *SDR* gene name, accession number and chromosome location

gene name	Accessible No.	Chr. Location	gene name	Accessible No.	Chr. Location
ak7	XP_029705918	16	*hsd17b7*	XP_029683692	20
ak7	XP_029688531	2	*hsd17b8*	XP_029700481	12
bdh1	XP_003966744	8	*hsd17b12*	XP_029702634	13
bdh1	XP_011606258	10	*hsd17b12*	XP_003967289	9
bdh2	XP_003971996	17	*hsd17b12*	XP_003971285	15
blvrb	XP_011607005	11	*hsd17b14*	XP_003965061	5
blvrb	XP_029685270	21	*hsdl1*	XP_029705857	16
c-factor	XP_029691043	4	*hsdl2*	XP_003965643	6
c-factor	XP_003964784	5	*htatip2*	XP_029702131	13
cbr1	XP_003963969	4	*kdsr*	XP_003977645	22
cbr4	XP_003973889	20	*mat2b*	XP_011609474	15
dcxr	XP_003978023	1	*ndufa9*	XP_003967219	9
decr1	XP_011603489	7	*nsdhl*	XP_003970530	14
decr2	XP_003961350	1	*pecr1*	XP_003976377	22
dhrs1	XP_011617211	22	*qdpra*	XP_003970718	14
dhrs3	XP_003973079	19	*rdh1*	XP_029692262	1
dhrs4	XP_011617463	8	*rdhe2*	XP_003975540	22
dhrs4	XP_029695380	8	*rdh5*	XP_003973175	19
dhrs7	XP_003964500	5	*rdh7*	XP_011604526	8
dhrs7	XP_003962718	2	*rdh7*	XP_011604524	8
dhrs7	XP_029692169	1	*rdh8*	XP_003978440	20
dhrs9	XP_011608290	1	*rdh8*	XP_003972354	17
dhrs11	XP_003968590	11	*rdh8*	XP_003964805	5
dhrs11	XP_029704433	15	*rdh10*	XP_003977403	10
dhrs11	XP_029688308	Un	*rdh10*	XP_003973294	19
dhrs12	XP_003971684	16	*rdh10*	XP_003973407	19
dhrs12	XP_011607073	11	*rdh11*	XP_003973611	19
dhrs13	XP_003962850	2	*rdh11*	XP_029698956	10
dhrs13	XP_003978473	11	*rdh11*	XP_003968203	10
dhrs13	XP_003978471	11	*rdh12*	XP_011615438	2
dhrsx	XP_003966447	8	*rdh12*	XP_011606133	10
dhrsx	XP_003962072	1	*rdh12*	XP_011606138	10
far1	XP_011605197	9	*rdh13*	XP_011601915	1
far1	XP_003972825	18	*rdh13*	XP_029694754	7
fasn	XP_011618161	1	*rdh14*	XP_003971836	16
gale	XP_003962464	2	*rdh14*	XP_003975230	21
gmds	XP_029684779	20	*sccpdh*	XP_003969518	13
hpgd	XP_003972133	17	*sdr39u1*	XP_003976184	22
hpgd	XP_029704076	15	*sdr42e1*	XP_011607874	13
hpgd	XP_011609662	15	*sdr42e2*	XP_029691907	5
hsd3b1	XP_029693476	1	*spr*	XP_003965138	6
hsd3b7	XP_003965001	5	*tdh*	XP_029688786	2
hsd11b1	XP_029684148	20	*tdh*	XP_003971710	16
hsd11b2	XP_003978403	13	*tgds*	XP_003966472	8

gene name	Accessible No.	Chr. Location	gene name	Accessible No.	Chr. Location
hsd17b1	XP_003964840	5	*tsta3*	XP_003974473	20
hsd17b2	XP_029697459	9	*tsta3*	XP_029684781	20
hsd17b3	XP_029685558	21	*uxs1*	XP_003961782	1
hsd17b4	XP_003974655	21	*wwox*	XP_003967177	9
hsd17b7	XP_003975860	22			

Table S10 Channel catfish (*Ictalurus punctatus*) *SDR* gene name, accession number and chromosome location

gene name	Accessible No.	Chr. Location	gene name	Accessible No.	Chr. Location
ak7	XP_017310927	25	*hsd17b12*	NP_001187450	27
ak7	XP_017331270	9	*hsd17b12*	XP_017314985	28
bdh1	XP_017336422	1	*hsd17b12*	XP_017341652	14
bdh2	XP_017316277	29	*hsd17b14*	XP_017347923	2
blvrb	XP_017321876	X	*hsdl1*	NP_001188103	25
c-factor	XP_017321931	X	*hsdl2*	NP_001188109	16
c-factor	XP_017329222	8	*htatip2*	NP_001187581	10
cbr1	XP_017312829	26	*kdsr*	XP_017327046	7
cbr4	XP_017310850	25	*mat2b*	NP_001188160	18
dcxr	XP_017338501	13	*ndufa9*	NP_001187320	8
decr1	XP_017332981	1	*nsdhl*	XP_017329728	8
decr2	XP_017321877	1	*pecr*	XP_017314584	27
dhrs3	XP_017306348	21	*qdpr*	NP_001187412	29
dhrs4	NP_001187298	7	*rdhe2*	XP_017328264	7
dhrs7a	XP_017319026	3	*rdh5*	XP_017352012	21
dhrs7b	XP_017316868	2	*rdh7*	XP_017333260	10
dhrs7c	XP_017338726	13	*rdh8*	XP_017310647	24
dhrs11	XP_017316187	28	*rdh8*	XP_017309850	24
dhrs12	XP_017334916	11	*rdh8*	XP_017313258	26
dhrs12	XP_017346919	17	*rdh10*	XP_017308487	23
dhrs13	XP_017344301	16	*rdh10*	XP_017351022	20
dhrs13	XP_017345624	17	*rdh11*	XP_017319503	3
dhrs13	XP_017346173	17	*rdh11*	XP_017347606	18
dhrsx	XP_017313692	26	*rdh11*	XP_017309982	24
dhrsx	XP_017326228	6	*rdh11*	XP_017306972	22
far1	XP_017349256	19	*rdh12*	XP_017328384	7
far2	XP_017330297	8	*rdh12*	XP_017333108	1
fasn	XP_017339235	12	*rdh13*	NP_001187312	1
gale	XP_017330774	9	*rdh13*	XP_017350741	1
gmds	XP_017311522	25	*rdh14*	XP_017320234	3
hpgd	XP_017318874	3	*rdh14*	XP_017311109	25
hpgd	XP_017347981	18	*sccpdh*	XP_017332497	9
hsd3b1	NP_001187004	6	*sdr39u1*	NP_001187350	7
hsd3b7	XP_017350880	2	*sdr42e1*	XP_017322269	X
hsd11b1	XP_017334213	10	*sdr42e2*	XP_017309543	24
hsd11b2	XP_017314777	27	*spra*	NP_001188016	28
hsd17b1	XP_017341929	2	*tdh*	XP_017310831	25
hsd17b2	XP_017341673	14	*tdh*	XP_017331113	9
hsd17b3	XP_017323206	5	*tgds*	XP_017337801	12
hsd17b4	XP_017323241	5	*tsta3*	XP_017312767	2
hsd17b7	XP_017335299	11	*uxs1*	XP_017326106	6
hsd17b10	XP_017341775	15	*wwox*	XP_017340558	14

Table S11 Zebrafish (*Danio rerio*) *SDR* gene name, accession number and chromosome location

Gene name	Accession No.	Chr. location	Gene name	Accession No.	Chr. location
ak7-a	NP_001166036	20	*hsd17b2*	XP_005163234	25
ak7-b	NP_001103168	17	*hsd17b3*	NP_956658	8
bdh1-a	NP_001082978	24	*hsd17b4*	NP_956430	8
bdh2	NP_001017809	1	*hsd17b7*	NP_001070796	6
blvrb-b	NP_001002686	18	*hsd17b8*	NP_001005292	19
c-factor-b	NP_956847	17	*hsd17b10*	NP_001006098	23
c-factor-a	NP_001074098	18	*hsd17b12b*	NP_001373557	5
cbr1-a	NP_919387	1	*hsd17b12a-b*	NP_955907	7
cbr1-b	NP_919360	21	*hsd17b12a-a*	NP_957175	25
cbr4	NP_991219	20	*hsd17b14*	NP_001003521	3
dcxr	NP_001017619	12	*hsdl1*	NP_001008607	20
decr1	NP_001002444	16	*hsdl2*	NP_955893	10
decr2	XP_005162937	24	*htatip2*	NP_571756	7
dhrs1	NP_001002205	2	*kdsr*	NP_957433	2
dhrs3	NP_001006070	11	*mat2b*	NP_001013492	21
dhrs3	NP_001003477	23	*ndufa9*	NP_001013477	25
dhrs4	NP_956861	7	*nsdhl*	NP_001017674	14
dhrs7c-a	XP_021335788	12	*pecr*	NP_001017727	11
dhrs7c-b	NP_001013557	3	*qdpra*	NP_001103939	14
dhrs7a	NP_001038811	13	*rdhe2*	NP_998043	2
dhrs7b	XP_021326304	3	*rdh5*	XP_005174018	22
dhrs9	NP_955903	9	*rdh8a-a*	XP_005163825	3
dhrs11-b	NP_001002143	15	*rdh8a-b*	NP_957082	1
dhrs11-a1	XP_005157476	15	*rdh8b*	NP_957001	3
dhrs11-a2	NP_001093518	5	*rdh10*	NP_001074052	24
dhrs12b-b	XP_002660947	2	*rdh10*	XP_021332944	6
dhrs12b-a	NP_987120.1	15	*rdh12c*	NP_001009912	24
dhrs12a	NP_001070025	9	*rdh12a*	XP_021328703	16
dhrs13	NP_001004641	10	*rdh12b*	NP_001002325	13
dhrs13	NP_001007425	15	*rdh13*	NP_001038920	19
dhrs13	NP_001003510	15	*rdh14b-b*	NP_001040655	20
dhrsx	XP_009290326	1	*rdh14a*	NP_001006031	13
far1	NP_001076558	4	*sccpdh*	NP_001005574	20
far2	XP_005174372	25	*sdr39u1*	NP_001070046	2
fasn	XP_009305081	12	*sdr42e1*	NP_001297010	18
gale	NP_001035389	17	*sdr42e2*	XP_003198236	3
gmds	NP_001095945	20	*spr*	NP_001122262	8
hpgd-b	NP_001313470	1	*tdh*	NP_998410	20
hsd3b1	XP_694204	9	*tgds*	NP_956111	6
hsd3b2	NP_997962	20	*tsta3*	NP_001008620	20
hsd3b7	NP_956103	3	*uxs1*	NP_775349	9
hsd11b1b	XP_009297199	2	*vcr*	NP_001005597	5
hsd11b2	NP_997885	7	*wwox*	NP_957207	25
hsd17b1	NP_991147	3			

Table S12 Spotted gar (*Lepisosteus oculatus*) S*DR* gene name, accession number and chromosome location

Gene name	Accession No.	Chr. location	Gene name	Accession No.	Chr. location
ak7	XP_006632319	7	*hsd17b12a*	XP_006642505	27
bdh1	XP_015216679	14	*hsd17b12b*	XP_006627459	2
bdh1	XP_015213190	1	*hsdl1*	XP_015213926	1
bdh2	XP_006629863	4	*hsdl2*	XP_006626695	2
c-factora-1	XP_006642530	27	*htatip2*	XP_006642700	27
c-factora-2	XP_006642538	27	*kdsr*	XP_006634509	9
cbr1	XP_006639155	17	*mat2b*	XP_006632013	6
cbr4	XP_006629992	4	*nmral*	XP_015213790	12
blvrb	XP_006627645	2	*nsdhl*	XP_006632910	7
hsd17b1	XP_006638267.1	15	*pecr*	XP_006636718	12
tgds	XP_006639064	17	*qdpra*	XP_006629235	4
hsd3b1	XP_006639419	17	*rdhe2b*	XP_015209198	9
hsd11b1	XP_006640112	19	*rdhe2a*	XP_006625820	1
dcxr	XP_006635201	10	*rdh3*	XP_006636595	12
decr1	XP_006636065	11	*rdh5*	XP_006629411	4
decr2	XP_006637144	13	*rdh7*	XP_006636668	12
dhrs1	XP_006643378	Un	*rdh8c*	XP_006631636	6
dhrs3	XP_006641990	25	*rdh8d*	XP_006634760	10
dhrs4	XP_006643379	Un	*rdh8b*	XP_006638429	15
dhrs7a	XP_006632385	7	*rdh8a*	XP_006631542	6
dhrs7c	XP_006635376	10	*rdh10*	XP_006633950	9
dhrs7b	XP_006637081	13	*rdh11*	XP_015206674	7
dhrs11	XP_006640902	22	*rdh12*	XP_006629038	3
dhrs12a	XP_006637642	14	*rdh12*	XP_006632753	7
dhrs12b	XP_006639385	17	*rdh12b*	XP_006634549	9
dhrs13	XP_006640955	22	*rdh12*	XP_006626755	2
dhrsx	XP_006638079	14	*rdh13*	XP_006641820	24
far1	XP_006642573	27	*rdh13*	XP_006638612	16
far2	XP_015207363	8	*rdh14*	XP_006626408	1
fasn	XP_006635198	10	*rdh14*	XP_006640365	20
gale	XP_006631280	6	*sccpdh*	XP_015206961	1
gmds	XP_015210171	9	*sdr39u1*	XP_006643365	Un
hpgd	XP_006630023	4	*sdr42e1*	XP_006641223	23
hpgd	XP_006632167	6	*sdr42e2*	XP_006637408	13
hsd3b7	XP_006630300	5	*spr*	XP_006626559	2
hsd11b2	XP_006641583	23	*tdh*	XP_006626147	1
hsd17b2	XP_015223426	23	*tdh2*	XP_006631419	6
hsd17b3	XP_006627025	2	*tsta3*	XP_006634231	9
hsd17b4	XP_015222862	2	*uxs1*	XP_015219916	17
hsd17b7	XP_006634994	10	*wwox*	XP_006641198	23

Table S13 Coelacanth (*Latimeria chalumnae*) S*DR* gene name, accession number and chromosome location

Gene name	Accession No.	Chr. location	Gene name	Accession No.	Chr. location
ak7	XP_005990373	Un	*hsd17b8*	XP_006001028	Un
bdh1	XP_005989438	Un	*hsd17b10*	XP_005997373	Un
bdh1	XP_005989441	Un	*hsd17b12b*	XP_005999877	Un
bdh2	XP_006009732	Un	*hsd17b12a*	XP_005986970	Un
blvrb	XP_005991674	Un	*hsd17b13*	XP_006013534	Un
c-factor	XP_006010560	Un	*hsd17b14*	XP_006010041	Un
cbr1	XP_005987953	Un	*hsdl1*	XP_006005871	Un
cbr1-a	XP_005998522	Un	*hsdl2*	XP_005993564	Un
cbr4	XP_006007376	Un	*htatip2*	XP_005997348	Un
dcxr	XP_005989131	Un	*kdsr*	XP_006009703	Un
decr1	XP_005990735	Un	*ndufa9*	XP_005989345	Un
decr2	XP_006008584	Un	*nmral*	XP_014349682	Un
dhrs1	XP_005998950	Un	*nsdh1*	XP_005994779	Un
dhrs3	XP_006005249	Un	*mat2b*	XP_005996910	Un
dhrs4	XP_005996001	Un	*pecr*	XP_006004352	Un
dhrs7a	XP_005986487	Un	*qdpra*	XP_005990483	Un
dhrs7c	XP_006006679	Un	*rdh3*	XP_005986247	Un
dhrs7b	XP_006006984	Un	*rdh5*	XP_005997700	Un
dhrs9	XP_005997700	Un	*rdh7*	XP_006013055	Un
dhrs11	XP_005986890	Un	*rdh8c*	XP_005991839	Un
dhrs12	XP_006013133	Un	*rdh8b*	XP_005994398	Un
dhrs13	XP_006002401	Un	*rdh8a*	XP_005994090	Un
dhrsx	XP_005999956	Un	*rdh10*	XP_006004567	Un
dhrsx	XP_014346183	Un	*rdh11*	XP_006012622	Un
far1	XP_006003555	Un	*rdh12a*	XP_005991563	Un
fasn	XP_005989172	Un	*rdh12b*	XP_014340281	Un
gale	XP_005988808.1	Un	*rdh13*	XP_006003247	Un
gmds	XP_006001192	Un	*rdh14*	XP_006011723	Un
hpgd	XP_005988376	Un	*rdh14*	XP_006007274	Un
hsd3b1	XP_006000122	Un	*sccpdh*	XP_005992887	Un
hsd3b7	XP_005988677	Un	*sdr39u1*	XP_006005390	Un
hsd3b7	XP_006002255	Un	*sdr42e1*	XP_006011120	Un
hsd11b1a	XP_014351335	Un	*sdr42e2*	XP_006004682	Un
hsd11b1b-2	XP_005987758	Un	*spra*	XP_005997709	Un
hsd11b1b-1	XP_006008167	Un	*tdh*	XP_006003046	Un
hsd11b2	XP_006011815	Un	*tdh2*	XP_006003314	Un
hsd17b1	XP_006004321	Un	*tgds*	XP_006005503	Un
hsd17b2	XP_014353240	Un	*tsta3*	XP_006011829	Un
hsd17b3	XP_006001958	Un	*uxs1*	XP_005998073	Un
hsd17b4	XP_006010793	Un	*wwox*	XP_005997682	Un
hsd17b7	XP_005994841	Un			

Table S14 Elephant shark (*Callorhinchus milii*)*SDR* gene name, accession number and chromosome location

Gene name	Accession No.	Chr. location	Gene name	Accession No.	Chr. location
ak7	XP_007904207	Un	*hsd17b4*	XP_007895547	Un
bdh1	XP_007895720	Un	*hsd17b7*	NP_001279038	Un
bdh2	XP_007890068	Un	*hsd17b12*	XP_007885841	Un
blvrb	XP_007884366	Un	*hsdl1*	NP_001279042	Un
rdh12a	XP_007905812	Un	*hsdl2*	NP_001279740	Un
rdh12b	XP_007886268	Un	*htatip2*	XP_007885883	Un
c-factor	XP_007900418	Un	*kdsr*	XP_007898714	Un
cbr1-a	XP_007904143	Un	*mat2b*	XP_007906302	Un
cbr1	NP_001279238	Un	*nmral*	XP_007893877	Un
dcxr	NP_001279223	Un	*nsdhl*	XP_007889646	Un
ndufa9	NP_001279619	Un	*pecr1*	XP_007908523	Un
decr1	XP_007901383	Un	*qdpra*	XP_007887212	Un
decr2	XP_007891877	Un	*rdhe2*	XP_007889201	Un
dhrs1	XP_007884759	Un	*rdh5*	XP_007894885	Un
dhrs3	XP_007883066	Un	*rdh7*	XP_007888076	Un
dhrs4	XP_007903043	Un	*rdh7*	XP_007888075	Un
dhrs7c	XP_007887026	Un	*rdh8a*	XP_007910403	Un
dhrs7a	XP_007902073	Un	*rdh8c*	XP_007885331	Un
dhrs7b	XP_007901680	Un	*rdh10*	XP_007885177	Un
dhrs9	XP_007888071	Un	*rdh11*	XP_007905811	Un
dhrs11	XP_007894794	Un	*rdh11*	XP_007910497	Un
dhrs12	XP_007883419	Un	*rdh11*	XP_007885177	Un
dhrs13	XP_007898985	Un	*rdh13*	XP_007892576	Un
dhrsx	NP_001279490	Un	*rdh14*	XP_007898595	Un
far1	XP_007885998	Un	*sccpdh*	XP_007896598	Un
fasn	XP_007886730	Un	*sdr42e1*	XP_007887456	Un
gale	XP_007906950	Un	*sdr42e2*	XP_007891999	Un
gmds	XP_007906885	Un	*spr*	XP_007906754	Un
hpgd	XP_007894130	Un	*syt12*	XP_007902992	Un
hsd3b1	XP_007894069	Un	*tdh2*	XP_007902650	Un
hsd11b1b	XP_007897756	Un	*tgds*	XP_007888980	Un
hsd11b1a	XP_007896936	Un	*tsta3*	XP_007893257	Un
hsd17b2	XP_007909924	Un	*uxs1*	XP_007883236	Un
hsd17b3	NP_001279925	Un	*wwox*	XP_007887397	Un

Table S15 Human (*Homo sapiens*) *SDR* gene name, accession number and chromosome location

Gene name	Accession No.	Chr. location	Gene name	Accession No.	Chr. location
ak7	NP_689540	14	*hsd17b3*	NP_000188	9
bdh1	NP_004484	X	*hsd17b4*	NP_000405	5
bdh1	XP_011511370	3	*hsd17b6*	NP_003716	12
bdh2	NP_064524	4	*hsd17b7*	NP_057455	1
blvrb	NP_000704	19	*hsd17b8*	NP_055049	Un
cbr1	NP_001748	21	*hsd17b10*	NP_004484	X
cbr3	NP_001227	21	*hsd17b11*	NP_057329	4
cbr4	NP_116172	4	*hsd17b12*	NP_057226	11
dcxr	NP_057370	17	*hsd17b13*	NP_835236	4
decr1	XP_011515182	8	*hsd17b14*	NP_057330	18
decr2	NP_065715	16	*hsdl1*	NP_113651	16
dhrs1	NP_612461	14	*hsdl2*	NP_115679	9
dhrs2	NP_005785	14	*htatip2*	NP_006401	11
dhrs3	NP_004744	1	*kdsr*	XP_006722496	18
dhrs4-1	NP_066284	Un	*ndufa9*	NP_004993	12
dhrs4-3	XP_006720309	14	*nmral*	NP_001292070	16
dhrs4-2	XP_011534988	14	*mat2b*	NP_877725	5
dhrs7a	NP_057113	14	*nsdhl*	NP_057006	X
dhrs7c	NP_001207422	17	*pecr1*	NP_060911	2
dhrs7b	NP_056325	17	*qdpra*	NP_001293069	4
dhrs9	NP_001276692	2	*rdhe2*	NP_620419	8
dhrs11	NP_077284	17	*rdh5*	NP_002896	12
dhrs12	NP_001257353	13	*rdh8*	EAW84062	19
dhrs13	NP_653284	17	*rdh10*	NP_742034	8
dhrsx	NP_660160	X	*rdh11*	NP_057110	14
far1	NP_115604	11	*rdh12*	NP_689656	14
far2	NP_060569	12	*rdh13*	NP_001139443	19
fasn	NP_004095	17	*rdh14a*	NP_065956	2
gale	NP_001008217	1	*rdh14b*	NP_001186032	3
gmds	NP_001491	6	*rdh16*	NP_003699	12
hpgd	NP_000851	4	*sccpdh*	NP_057086	1
hsd3b1	NP_000853	1	*sdr42e2*	XP_011506928	16
hsd3b7-1	NP_079469	16	*sdr42e1*	NP_660151	16
hsd3b7-2	XP_005255658	16	*syt12*	XP_011543648	11
hsd11b1a	NP_005516	1	*tgds*	NP_055120	13
hsd11b1b	NP_940935	19	*tsta3*	NP_003304	8
hsd11b2	NP_000187	16	*uxs1*	NP_079352	2
hsd17b1	NP_000404	17	*wwox*	NP_057457	16
hsd17b2	NP_002144	16			

Table S16 Tropical clawed frog (*Xenopus tropicalis*) S*DR* gene name, accession number and chromosome location

Gene name	Accession No.	Chr. location	Gene name	Accession No.	Chr. location
ak7	NP_001011352	8	*hsd17b11*	NP_001011304	1
bdh1	XP_002937888	5	*hsd17b12*	XP_002941866	3
bdh1	XP_002937878	5	*hsd17b12*	NP_001017234	4
bdh2	XP_002934803	1	*hsd17b13*	NP_001011240	1
blvrb	XP_012823332	8	*hsd17b14*	XP_002935043	7
cbr1	NP_001011190	2	*hsdl1*	XP_002937365	4
cbr1	NP_001120040	2	*hsdl2*	NP_001008673	1
cbr1	XP_004912181	2	*htatip2*	XP_031755863	4
cbr4	NP_001007873	1	*kdsr*	NP_001072722	6
dcxr	NP_989100	10	*mat2b*	NP_001016828	3
decr1	NP_001076813	6	*ndufa9*	NP_001011432	3
decr2	XP_002932500	9	*nmral*	NP_001016962	9
dhrs3	NP_001008431	7	*nsdhl*	NP_001017290	8
dhrs4	NP_001015784	1	*pecr*	XP_031749362	9
dhrs7a	NP_001015708	8	*qdpra*	NP_001011492	1
dhrs7b	NP_001072246	9	*rdh2*	NP_001039196	3
dhrs7c	NP_001107423	10	*rdhe2*	XP_012819978	6
dhrs9	NP_001001191	9	*rdh5*	NP_001011363	2
dhrs11	XP_012816495	2	*rdh7*	XP_017947370	2
dhrs12	NP_001072547	2	*rdh7*	NP_001107972	2
dhrs13	NP_989311	2	*rdh7*	NP_001017189	2
dhrsx	XP_004911797	2	*rdh8*	XP_031755239	3
far1	XP_017948360	4	*rdh8*	XP_002940417	10
fasn	XP_031750124	10	*rdh10*	NP_001011091	6
gale	NP_001006762	2	*rdh10*	XP_002933209	10
gmds	NP_001032330	6	*rdh11*	XP_031751072	1
hpgd	NP_001007992	1	*rdh12*	XP_031747877	1
hsd3b2	XP_031752287	2	*rdh13*	NP_001011000	5
hsd3b7	NP_001120501	9	*rdh14*	XP_004910666	1
hsd3b7	XP_002935341	2	*rdh14*	NP_001017231	5
hsd11b1	NP_001090784	1	*rdh16*	NP_001107359	2
hsd11b1	XP_004910661	1	*sccpdh*	XP_002931533	5
hsd11b1	XP_031752637	2	*sdr39u1*	NP_001016261	1
hsd17b1	XP_002935954	3	*sdr42e1*	XP_002934995	4
hsd17b2	XP_002934996	4	*sdr42e2*	XP_004918342	9
hsd11b2	XP_002937366	4	*spr*	NP_001120067	1
hsd17b3	XP_012827001	1	*tdh*	XP_004915002	5
hsd17b4	NP_001027490	1	*tgds*	XP_002939327	2
hsd17b6	XP_002939751	2	*tsta3*	NP_001032328	6
hsd17b7	NP_001016209	4	*uxs1*	NP_001006849	2
hsd17b8	NP_001016671	8	*wwox*	XP_017948987	4
hsd17b10	NP_001016511	8			

Table S17 Chicken (*Gallus gallus*) *SDR* gene name, accession number and chromosome location

Gene name	Accession No.	Chr. location	Gene name	Accession No.	Chr. location
ak7	XP_426462	5	*hsd17b10*	XP_015128617	Un
bdh1	NP_001006547	9	*hsd17b11*	XP_420547	4
bdh1	XP_015147086	9	*hsd17b11*	XP_426310	4
bdh1	XP_004935295	3	*hsd17b12*	XP_015142684	5
bdh2	XP_015141101	4	*hsd17b14*	XP_015148724	12
blvrb	NP_990721	Un	*htatip2*	XP_422206	8
c-factor-1	NP_001305767	11	*hsdl1*	NP_001005837	11
c-factor-2	NP_001305772	11	*hsdl2*	XP_003643140	Z
cbr1	XP_015154922	1	*kdsr*	XP_015137662	2
cbr4	XP_004940989	4	*mat2b*	XP_015149155	13
dcxr	NP_989556	28	*ndufa9*	NP_001006281	1
decr1	XP_418328	2	*nmral*	NP_001025816	14
decr2	XP_003642207	14	*nmral*	XP_420279	4
dhrs3	NP_001264839	21	*pecr1*	NP_001006522	7
dhrs4	NP_001264054	1	*qdpra*	NP_001006566	4
dhrs7a	XP_421423	5	*sccpdh*	NP_001012893	3
dhrs7b	XP_004945233	14	*sdr42e1*	XP_004944316	11
dhrs7c	NP_001264924	18	*sdr42e2*	NP_001182086	14
dhrs9	XP_422015	7	*sdr42e2*	XP_015150041	14
dhrs11	NP_989838	19	*spr*	XP_423038	4
dhrs12	XP_001233773	10	*syt12*	XP_004941538	5
dhrs13	XP_003642478	19	*tdh*	XP_420039	3
dhrsx	XP_015133220	1	*tgds*	XP_416988	1
far1	NP_001026350	5	*tsta3*	NP_001264377	2
far2	XP_024998241	1	*rdh1*	XP_015135719	7
fasn	NP_990486	18	*rdh2*	NP_990326	10
gale	XP_417833	23	*rdhe2*	NP_001026193	2
gmds	XP_015137673	2	*rdhe2*	XP_015138173	2
hpgd	NP_001264713	4	*rdh5*	NP_990044	33
hsd3b1	NP_990449	1	*rdh7*	XP_015155884	33
hsd3b7	XP_415754	19	*rdh8c*	XP_025008725	6
hsd11b1a	NP_001001201	28	*rdh8b*	XP_003642855	27
hsd11b1b	XP_001235138	26	*rdh8a*	XP_015129530.2	30
hsd11b1	XP_417988	26	*rdh10*	NP_001186388	2
hsd11b2	XP_003641938	11	*rdh12a*	XP_421193	5
hsd17b1	NP_990168	27	*rdh12b*	XP_025006229	4
hsd17b2	XP_414168	11	*rdh14*	XP_015140557	3
hsd17b3	XP_425046	7	*rdh16*	XP_015155883	33
hsd17b4	NP_990274	7	*uxs1*	XP_416926	1
hsd17b7	NP_001264435	8	*wwox*	NP_001025745	11

Table S18 Python (*Python bivittatus*) *SDR* gene name, accession number and chromosome location

Gene name	Accession No.	Chr. location	Gene name	Accession No.	Chr. location
dhrs13	XP_025033223	Un	*hsdl1*	XP_007443016	Un
sdr42e1	XP_007433144	Un	*hsd17b12*	XP_007429327	Un
sdr42e2	XP_025020383	Un	*hsd17b3*	XP_007439207	Un
sdr42e2	XP_025020384	Un	*mat2b*	XP_007435624	Un
hsd3b2	XP_007441584	Un	*hsd17b10*	XP_007425791	Un
nsdhl	XP_007435121	Un	*gmds*	XP_007431194	Un
hsd3b7	XP_007434134	Un	*dhrs1*	XP_007442487	Un
decr2	XP_007436493	Un	*decr1*	XP_007441340	Un
far1	XP_025029052	Un	*pecr1*	XP_007420219	Un
far1	XP_025033269	Un	*kdsr*	XP_015743296	Un
far2	XP_025033051	Un	*hsdl2*	XP_007427284	Un
hsd17b6	XP_007442413	Un	*dhrs3*	XP_025027210	Un
rdh5	XP_007435496	Un	*dhrs11*	XP_007441394	Un
dhrs9	XP_015745681	Un	*nmral*	XP_007445572	Un
rdh16	XP_025032955	Un	*hsd17b11*	XP_007441381	Un
rdh16	XP_007421382	Un	*bdh2*	XP_007429867	Un
rdh16	XP_025033208	Un	*cbr1*	XP_007423386.1	Un
rdh16	XP_025033353	Un	*dhrs7a*	XP_007429182	Un
hsd11b2	XP_025031993	Un	*dhrs7b*	XP_007428301	Un
hsd17b2	XP_025025501	Un	*dhrs7c*	XP_007445065	Un
hsd17b6	XP_025033360	Un	*rdh12a*	XP_025033230	Un
hsd17b6	XP_025033359	Un	*rdh12b*	XP_025029413	Un
rdh2	XP_007445192	Un	*rdh8a-a*	XP_007427788	Un
hsd17b14	XP_007442980	Un	*rdh8b*	XP_007433595	Un
dhrs4	XP_007437370	Un	*rdh11*	XP_007445238	Un
bdh2	XP_007429867	Un	*rdh11*	XP_007445295	Un
cbr4	XP_025023466	Un	*rdh11*	XP_025033337	Un
hsd17b4	XP_007424211	Un	*rdh10*	XP_025020282	Un
dhrs9	XP_007421904	Un	*rdh14*	XP_007421740	Un
pecr	XP_007420219	Un	*hpgd*	XP_007432129	Un
rdhe2	XP_007430283	Un	*tsta3*	XP_007444506	Un
wwox	XP_025018764	Un	*dcxr*	XP_007434254	Un
qdpra	XP_007443182	Un	*blvrb*	XP_025026008	Un
nsdhl	XP_007435121	Un	*hsd11b1a*	XP_007438759	Un
gale	XP_007430572	Un	*hsd11b1b*	XP_007440169	Un
tdh	XP_025027957	Un	*uxs1*	XP_007437309	Un
ndufa9	XP_025027221	Un	*uxs1*	XP_007439738	Un
sdr39u1	XP_007438905	Un	*bdh1*	XP_025032716	Un
hsd17b7	XP_007439378	Un	*bdh1*	XP_025032396	Un
spr	XP_007431552	Un	*c-factor*	XP_025021897	Un
fasn	XP_007434239	Un	*c-factor*	XP_007427400	Un
htatip2	XP_007440617	Un	*c-factor*	XP_007440030	Un
sccpdh	XP_007433911	Un			

Table S19 Lamprey (*Lampetra japonicavase*) *SDR* gene name, accession number and chromosome location

Gene name	Accession No.	Chr. location	Gene name	Accession No.	Chr. location
rdh14	ENSPMAP00000011314	Un	*bdh2*	ENSPMAP00000004475	Un
rdh13	ENSPMAP00000010018	Un	*decr2*	ENSPMAP00000001149	Un
rdh12	ENSPMAP00000004718	Un	*decr1*	ENSPMAP00000002173	Un
dhrsx	ENSPMAP00000004429	Un	*hsd17b12b*	ENSPMAP00000009403	Un
dhrs12	ENSPMAP00000000645	Un	*bdh1*	ENSPMAP00000004569	Un
cbr1	ENSPMAP00000007590	Un	*sdr42e2*	ENSPMAP00000007879	Un
hsd17b7	ENSPMAP00000008339	Un	*nsdh1*	ENSPMAP00000010685	Un
dhrs7b	ENSPMAP00000005802	Un	*far1*	ENSPMAP00000008176	Un
rdh5	ENSPMAP00000001893	Un	*qdpr*	ENSPMAP00000003173	Un
dhrs4	ENSPMAP00000007754	Un	*tdh1*	ENSPMAP00000000557	Un
RDH16	ENSPMAP00000001876	Un	*ndufa9*	ENSPMAP00000010127	Un
dhrs1	ENSPMAP00000008147	Un	*sdr39u1*	ENSPMAP00000011409	Un
dhrs1	ENSPMAP00000003208	Un	*dhrs9*	ENSPMAP00000010678	Un
hsd12	ENSPMAP00000001246	Un	*tsta3*	ENSPMAP00000007696	Un
hsd17b4	ENSPMAP00000001746	Un	*fasn*	ENSPMAP00000000501	Un
kdsr	ENSPMAP00000004793	Un	*mat2b*	ENSPMAP00000009924	Un
pecr	ENSPMAP00000004814	Un	*hsd17B10*	ENSPMAP00000006346	Un

Table S20 Vase tunicate (*Ciona intestinalis*) *SDR* gene name, accession number and chromosome location

Gene name	Accession No.	Chr. location	Gene name	Accession No.	Chr. location
ak7	XP_018670747	Un	*hsd11b1*	XP_002131037	9
bdh1	XP_002128232	1	*hsd17b6*	XP_002121207	Un
bdh2	XP_002128839	Un	*hsd17b7*	XP_002130850	9
blvrb	XP_002128791	5	*hsd17b8*	XP_002129957	9
cbr1	XP_002129754	Un	*hsd17b10*	XP_002129755	8
decr2	XP_002131655	3	*hsd17b11*	XP_002120364	9
decr2	XP_002128418	3	*hsd17b12*	XP_018671855	Un
dcxr	XP_018666643	3	*hsd17b12*	XP_002130556	3
dhrs1	XP_009861303	Un	*hsdl1*	XP_002130269	14
dhrs1	XP_009858684	Un	*hsdl2*	XP_002120177	8
dhrs1	XP_026695379	Un	*kdsr*	XP_002127644	2
dhrs1	XP_002131026	Un	*ndufa9*	XP_002125647	Un
dhrs4	XP_002131419	2	*nmral*	XP_004226527	9
dhrs7	XP_002123111	11	*pecr*	XP_026694592	Un
dhrs11	XP_026692271	10	*qdpra*	XP_002128545	8
dhrs11	XP_002130258	10	*rdh2*	XP_026690052	4
dhrs11	XP_018669147	10	*rdhe2*	XP_002130984	Un
dhrs11	XP_018669275	10	*rdh11*	XP_026690134	4
dhrs11	XP_009859978	10	*rdh12*	XP_002127110	Un
dhrs11	XP_002130309	10	*rdh12*	XP_002132183	3
dhrs11	XP_009859973	10	*rdh13*	XP_026695697	2
dhrs12	XP_002132114	6	*rdh13*	XP_018669257	10
dhrs12	XP_002132115	6	*rdh14*	XP_002124144	1
dhrs13	XP_002130108	7	*rdh14*	XP_002130502	1
dhrs13	XP_002127240	1	*rdh14*	XP_002128635	1
far1	XP_002129857	12	*rdh14*	XP_009857533	2
far1	XP_009860455	12	*sccpdh*	XP_002125674	3
far1	XP_004226772	12	*sdr39u1*	XP_002127876	2
fasn	XP_018667271	4	*sdr42e1*	XP_026693735	Un
gale	XP_026695662	Un	*spra*	XP_002123470	1
gale	XP_002127539	5	*tdh*	XP_002129605	1
gmds	XP_002131034	13	*uxs1*	XP_002127542	2
hsd3b7	XP_002127579	Un	*uxs1*	XP_009859008	7
hsd3b7	XP_002127558	Un	*wwox*	XP_009860463	12
hsd11b1	XP_009859047	7			

Table S21 Black tiger shrimp (*Penaeus monodon*) *SDR* gene name, accession number and chromosome location

Gene name	Accession No.	Chr. location	Gene name	Accession No.	Chr. location
bdh2	XP_037789628	19	*rdh14*	XP_037785018	14
bdh1	XP_037783265	12	*ndufa9*	XP_037796808	29
bdh1	XP_037782916	12	*sdr39u1*	XP_037794897	26
bdh1	XP_037783029	12	*hpgd*	XP_037798053	31
bdh1	XP_037776201	5	*dhrs4*	XP_037785066	14
bdh1	XP_037783529	12	*hsd17b10*	XP_037790832	21
dhrs7	XP_037799787	34	*wwox*	XP_037793306	24
dhrs12	XP_037779040	1	*tsta3*	XP_037784933	14
dhrs12	XP_037784365	13	*mat2b*	XP_037782736	11
rdh13	XP_037784863	14	*spr*	XP_037796206	28
rdh13	XP_037777995	6	*dcxr*	XP_037785890	15
rdh13	XP_037780282	9	*dcxr*	XP_037785886	15
sdr42e1	XP_037774658	4	*fasn*	XP_037781418	10
gale	XP_037781386	10	*htatip2*	XP_037782542	11
decr1	XP_037785977	15	*bdh2*	XP_037795649	28
hsd17b8	XP_037789595	19	*sccpdh*	XP_037799598	34
hsd17b8	XP_037789592	19	*sccpdh*	XP_037787469	1
hsd17b8	XP_037789594	19	*blvrb*	XP_037772871	43
rdh12	XP_037777098	6	*blvrb*	XP_037790311	20
rdh12	XP_037785017	14	*hsdl1*	XP_037780763	9
rdh12	XP_037802662	39	*hsdl1*	XP_037780853	9
rdh11	XP_037799569	34	*hsdl1*	XP_037780757	9
rdh11	XP_037782589	11	*gmds*	XP_037773457	4
rdh11	XP_037777811	6	*hsd3b2*	XP_037780361	9
rdh11	XP_037777905	6	*hsd17b12*	XP_037798913	33
rdh11	XP_037777387	6	*pecr*	XP_037790963	21
dhrs11	XP_037795931	28	*uxs1*	XP_037778624	7
dhrs11	XP_037795937	28	*uxs1*	XP_037778626	7
qdpra	XP_037776464	1	*uxs1*	XP_037781385	10
rdhe2	XP_037800876	36	*dhrsx*	XP_037788525	17
rdhe2	XP_037800587	36	*kdsr*	XP_037801642	28
rdhe2	XP_037785991	15	*c-factor*	XP_037784449	13
rdhe2	XP_037781877	11	*c-factor*	XP_037799494	3
hsd17b4	XP_037780943	9	*cbr1*	XP_037790327	20
sdr9c7	XP_037782276	11	*rdh3*	XP_005986247	Un
tdh	XP_037796059	28			

Table S22 Domestic silkworm (*Bombyx mori*) *SDR* gene name, accession number and chromosome location

Gene name	Accession No.	Chr. location	Gene name	Accession No.	Chr. location
ak7	XP_021207455	22	*hpgd*	XP_004931596	11
bdh1	XP_037875230	22	*hsd17b8*	XP_037871730	15
blvrb	XP_037876243	25	*hsd17b11*	XP_004931746	3
c-factor	XP_004921586	10	*hsd17b12*	XP_004928860	16
cbr1	XP_004926634	27	*hsd17b13*	XP_004923881	5
cbr1	XP_004930987	4	*hsdl1*	XP_012548494	4
dcxr	NP_001040432	17	*kdsr*	XP_004932635	23
dcxr	XP_004925887	17	*ndufa9*	XP_004927924	13
dhrs4	XP_004931489	11	*qdpra*	XP_037873689	19
dhrs7	XP_004924837	10	*rdhe2*	XP_021202128	5
far1	XP_004930776	23	*rdh11*	XP_037870450	12
far1	XP_012545692	23	*rdh11*	XP_021203036	9
far1	XP_004930761	23	*rdh11*	XP_012545059	9
far1	XP_004925993	23	*rdh11*	XP_037868965	9
far1	XP_012549538	10	*rdh11*	XP_004932899	11
far1	XP_012545674	23	*rdh11*	XP_004928973	16
far1	XP_037867828	1	*rdh12*	XP_021209022	15
far1	XP_004925992	23	*rdh12*	XP_037874094	20
far1	XP_004930778	23	*rdh13*	XP_021209023	15
far1	XP_004929428	12	*rdh13*	XP_004925724	25
far1	XP_012544376	23	*rdh13*	XP_037874550	21
far1	XP_012544375	23	*rdh14*	XP_037868950	9
fasn	XP_037870080	11	*rdh14*	XP_037868902	9
fasn	XP_037872251	16	*sdr16c6*	XP_004931747	3
fasn	XP_037872252	16	*sdr16c6*	XP_012543911	5
fasn	XP_037872253	16	*sdr39u1*	XP_037869568	11
fasn	XP_037872513	16	*sccpdh*	XP_037867535	6
fasn	XP_037872512	16	*spr*	XP_004924416	3
fasn	XP_037872193	16	*tdh*	NP_001037542	4
fasn	XP_037873188	18	*tsta3*	XP_037867676	6
fasn	XP_037873187	18	*uxs1*	XP_004928459	15
gale	XP_004927696	20	*wwox*	XP_021206866	4
gmds	XP_004928173	18			

Table S23 Fruit fly (*Drosophila melanogaster*) *SDR* gene name, accession number and chromosome location

Gene name	Accession No.	Chr. location	Gene name	Accession No.	Chr. location
bdh2	NP_610724	2R	*hpgd*	NP_001285777	2L
blvrb	NP_001097729	3R	*hpgd*	NP_001027272	2L
c-factor	NP_001285012	X	*hsd17b2*	NP_651725	3R
cbr1	NP_001286630	2R	*hsd17b4*	NP_001285318	X
dcxr	NP_001259693	X	*hsd17b7*	NP_001287387	3R
dhrs3	NP_001033900	2L	*hsd17b7*	NP_001287387	3R
dhrs7	NP_608616	2L	*hsd17b8*	NP_001259199	X
dhrs7	NP_651717	3R	*hsd17b10*	NP_523396	X
dhrs11	NP_788887	X	*hsd17b12*	NP_572420	X
far1	NP_001163168	2R	*hsdl1*	NP_001097168	2L
far1	NP_726499	2R	*hsdl1*	NP_724023	2L
far1	NP_001247272	3R	*hsdl1*	NP_001285997	2L
far1	NP_610535	2R	*hsdl1*	NP_609817	2L
far1	NP_651652	3R	*hsdl2*	NP_651578	3R
far1	NP_001027183	3R	*kdsr*	NP_651363	3R
far1	NP_611140	2R	*ndufa9*	NP_001262116	3L
far1	NP_650568	3R	*qdpra*	NP_001014579	3L
far1	NP_001260257	2L	*rdh12*	NP_001287390	3R
far1	NP_650566	3R	*rdh12*	NP_610310	2R
far1	NP_001259264	X	*rdh12*	NP_001260784	2R
far1	NP_650567	3R	*rdh12*	NP_001260787	2R
far1	NP_001097509	3L	*rdh12*	NP_001260786	2R
far1	NP_001027182	3R	*rdhe2*	NP_001260655	2L
far1	NP_001286495	2R	*rdhe2*	NP_608859	2L
far1	NP_650848	3R	*sccpdh*	NP_649517	3R
fasn	NP_001137778	2L	*sccpdh*	NP_650190	3R
fasn	NP_647613	2L	*sdr39u1*	NP_610813	2R
fasn	NP_001015405	3L	*spra*	NP_727265	X
gmds	NP_608888	2L	*tdh*	NP_649230	3L
gale	NP_001246537	3L	*tsta3*	NP_611734	2R
hpgd	NP_648885	3L	*uxs1*	NP_648182	3L
hpgd	NP_652673	3L			

Table S24 Nematode (*Caenorhabditis Elegans*) *SDR* gene name, accession number and chromosome location

Gene name	Accession No.	Chr. location	Gene name	Accession No.	Chr. location
dcxr	NP_506182	V	*hsd17b12*	NP_506449	V
decr	NP_495805	II	*hsd17b12*	NP_507092	V
decr	NP_495714	II	*hsd17b13*	NP_492563	I
decr	NP_506413	V	*hsdl1*	NP_609817	2L
dhrs1	NP_498146	III	*hsdl2*	NP_001021972	II
dhrs1	NP_505327	V	*htatip2*	NP_001367614	II
dhrs1	NP_508580	X	*kdsr*	NP_001294377	IV
dhrs3	NP_509294	X	*ndufa9*	NP_497675	V
dhrs4	NP_506230	c	*qdpra*	NP_499760	III
dhrs7	NP_510793	X	*rdhe2*	NP_505915	V
dhrs7	NP_001257285	X	*rdhe2*	NP_001122508	I
dhrs9	NP_505941	V	*sccpdh*	NP_503577	V
dhrs9	NP_491575	I	*syt12*	NP_506407	V
dhrs9	NP_504554	V	*syt12*	NP_506406	V
dhrs12	NP_507860	V	*syt12*	NP_495520	II
far1	NP_001370741	X	*syt12*	NP_505920	V
fasn	NP_492417	I	*syt12*	NP_001024318	V
gale	NP_001021051	I	*syt12*	NP_505919	V
gmds	NP_493106	I	*syt12*	NP_501850	IV
gmds	NP_501564	IV	*syt12*	NP_505922	V
hsd3b1	NP_493402	I	*syt12*	NP_001255457	IV
hsd3b1	NP_508851	v	*syt12*	NP_505924	IV
hsd3b1	NP_508852	X	*syt12*	NP_505921	V
hsd17b1	NP_508591	X	*tdh*	NP_504433	V
hsd17b4	NP_509146	X	*tsta3*	NP_498540	III
hsd17b4	NP_495494	II	*uxs1*	NP_001040727	II
hsd17b7	NP_506570	V	*uxs1*	NP_501418	IV
hsd17b7	NP_491557	I	*vcr*	NP_505755	V
hsd17b8	NP_508282	X	*vcr*	NP_503751	V
hsd17b8	NP_499346	III	*wwox*	NP_495501	II
hsd17b10	NP_502083	IV	*wwox*	NP_503155	V
hsd17b12	NP_001254936	III	*wwox*	NP_495500	II
hsd17b12	NP_505205	V	*wwox*	NP_001368222	II

Table S25 Sponge (*Amphimedon queenslandica*) *SDR* gene name, accession number and chromosome location

Gene name	Accession No.	Chr. location	Gene name	Accession No.	Chr. location
ak7	XP_003385586	Un	*hpgd*	XP_003390271	Un
bdh2	XP_003384090	Un	*hpgd*	XP_003390268	Un
cbr1	XP_019850605	Un	*hpgd*	XP_019857499	Un
decr1	XP_003388595	Un	*kdsr*	XP_003386428	Un
decr2	XP_003384531	Un	*mat2b*	XP_003388343	Un
dcxr	XP_003384523	Un	*nsdhl*	XP_011406755	Un
dhrs4	XP_003382463	Un	*ndufa9*	XP_003390219	Un
dhrs7b	XP_003387672	Un	*nmral*	XP_003384294	Un
dhrs7	XP_003386866	Un	*pecr*	XP_019856223	Un
dhrs7	XP_019853259	Un	*qdpra*	XP_019849430	Un
dhrs11	XP_003389223	Un	*rdh11*	XP_019850139	Un
dhrs11	XP_011405955	Un	*rdh12*	XP_011404099	Un
dhrs11	XP_003382361	Un	*rdh12*	XP_019855897	Un
dhrs11	XP_003391359	Un	*rdh12*	XP_011410399	Un
dhrs11	XP_003384816	Un	*rdh12*	XP_003382417	Un
dhrs11	XP_003391683	Un	*rdh13*	XP_011404089	Un
dhrs12	XP_003383658	Un	*rdh13*	XP_019855896	Un
dhrsx	XP_019849023	Un	*rdh13*	XP_011403108	Un
far1	XP_003384945	Un	*rdh13*	XP_003385583	Un
far1	XP_019861508	Un	*rdhe2*	XP_011404092	Un
gmds	XP_003384422	Un	*sccpdh*	XP_003389768	Un
hsd11b1	XP_019852624	Un	*sdr42e1*	XP_019850393	Un
hsd17b1	XP_019858264	Un	*sdr16c6*	XP_003384351	Un
hsd17b4	XP_003382406	Un	*spr*	XP_003383589	Un
hsd17b7	XP_019849790	Un	*spr*	XP_003392127	Un
hsd17b10	XP_003385670	Un	*spr*	XP_003391716	Un
hsd17b11	XP_003384325	Un	*spr*	XP_003383562	Un
hsdl1	XP_011406972	Un	*spr*	XP_003383588	Un
hsdl1	XP_011408621	Un	*tsta3*	XP_019854374	Un
hsdl1	XP_003388318	Un	*uxs1*	XP_011407364	Un
hsdl2	XP_003387239	Un	*uxs1*	XP_003391988	Un
hpgd	XP_019858877	Un	*uxs1*	XP_003389948	Un
hpgd	XP_011407268	Un	*wwox*	XP_011406756	Un
hpgd	XP_019857508	Un			

Table S26 Paramecium *SDR* gene name, accession number and chromosome location

Gene name	Accession No.	Chr. location	Gene name	Accession No.	Chr. location
cbr1	XP_001428669	Un	*hsd17b1*	XP_001450369	Un
cbr4	XP_001428751	Un	*hsd17b3*	XP_001347171	Un
c-factor	XP_001424995	Un	*hsd17b3*	XP_001458027	Un
decr1	XP_001436183	Un	*hsd17b4*	XP_001457027	Un
decr1	XP_001433687	Un	*hsd17b4*	XP_001426526	Un
decr2	XP_001457066	Un	*hsd17b4*	XP_001455301	Un
dhrs1	XP_001435177	Un	*hsd17b4*	XP_001440682	Un
dhrs7	XP_001425465	Un	*hsd17b4*	XP_001433168	Un
dhrs7	XP_001424921	Un	*hsdl1*	XP_001346918	Un
dhrs12	XP_001458911	Un	*hsdl1*	XP_001458281	Un
dhrs12	XP_001428848	Un	*rdh5*	XP_001443482	Un
dhrs13	XP_001454452	Un	*rdh10*	XP_001444041	Un
dhrsx	XP_001444470	Un	*rdh10*	XP_001448133	Un
far1	XP_001424995	Un	*rdh12*	XP_001461235	Un
hsd3b1	XP_001451145	Un	*rdh12*	XP_001427169	Un
hsd11b1	XP_001439510	Un	*rdh12*	XP_001430409	Un
hsd17b1	XP_001430965	Un	*rdh14*	XP_001446448	Un

Table S27 Subfamily designation and copy number of each SDR gene in tilapia, spotted gar and human

Gene	Abbreviation	Subfamily designation	Tilapia	Spotted gar	Human
UDP-glucose 4-epimerase-like	gale	SDR1E	1	1	1
dTDP-D-glucose 4,6-dehydratase	tgds	SDR2E	1	1	1
GDP-mannose 4,6 dehydratase	gmds	SDR3E	1	1	1
GDP-L-fucose synthase	*tsta3*	SDR4E	1	1	1
3-hydroxyacyl-CoA dehydrogenase	*hsd17b10*	SDR5C	1	1	1
UDP-glucuronic acid decarboxylase	*uxs1*	SDR6E	1	1	1
retinol dehydrogenase 11	*rdh11*	SDR7C	2	1	1
retinol dehydrogenase 12	*rdh12*	SDR7C	4	1	1
retinol dehydrogenase 13	*rdh13*	SDR7C	2	1	1
retinol dehydrogenase 14	*rdh14*	SDR7C	3	1	1
dehydrogenase/reductase SDR family member 13	*dhrs13*	SDR7C	3	1	1
dehydrogenase/reductase SDR family member on chromosome X	*dhrsx1*	SDR7C	2	2	2
Peroxisomal multifunctional enzyme	*hsd17b4*	SDR8C	1	1	1
11-beta-dehydrogenase 2	*hsd11b2*	SDR9C	1	1	1
dehydrogenase/reductase SDR family member 9	*dhrs9*	SDR9C	2	1	0
retinol dehydrogenase 3	*rdh3*	SDR9C	2	1	0
11-cis retinol dehydrogenase	*rdh5*	SDR9C	1	1	1
retinol dehydrogenase 7	*rdh7*	SDR9C	1	1	0
D-beta-hydroxybutyrate dehydrogenase, mitochondrial	*bdh1*	SDR9C	2	2	2
fatty acyl-CoA reductase	*far*	SDR10E	2	2	2
3beta-hydroxysteroid dehydrogenase type 1	*hsd3b1*	SDR11E	1	1	1
3beta-hydroxysteroid dehydrogenase type 2	*hsd3b2*	SDR11E	0	1	1
3beta-hydroxysteroid dehydrogenase type 7	*hsd3b7*	SDR11E	1	1	1
17-beta hydroxysteroid dehydrogenase type 12	*hsd17b12*	SDR12C	3	2	1
17-beta-hydroxysteroid dehydrogenase 3	*hsd17b3*	SDR12C	1	1	1
hydroxysteroid dehydrogenase 1	*hsdl1*	SDR12C	1	1	1
hydroxysteroid dehydrogenase 2	*hsdl2*	SDR13C	1	1	1
L-threonine 3-dehydrogenase	*tdh1*	SDR14E	3	2	0
3-hydroxybutyrate dehydrogenase type 2	*bdh2*	SDR15C	1	1	0
retinol dehydrogenase 10-A	*rdh10*	SDR16C	3	1	1
epidermal retinol dehydrogenase 2	*rdhe2*	SDR16C	1	1	1
short-chain dehydrogenase/reductase 3	*dhrs3*	SDR16C	1	1	1
2,4-dienoyl-CoA reductase peroxisomal	*decr2*	SDR17C	1	1	1
2,4-dienoyl-CoA reductase peroxisomal	*decr*	SDR18C	1	1	1
dehydrogenase/reductase SDR family member 1	*dhrs1*	SDR19C	1	1	1
L-xylulose reductase	*dexr*	SDR20C	1	1	1
carbonyl reductase	*cbr1*	SDR21C	4	1	1
NADH dehydrogenase 1 alpha subcomplex, 9	*ndufa9*	SDR22E	1	1	1
methionine adenosyltransferase 2	*mat2b*	SDR23E	1	1	1
dehydrogenase/reductase SDR family member 11	*dhrs11*	SDR24C	14	1	1
dehydrogenase/reductase SDR family member 4	*dhrs4*	SDR25C	1	1	1

Gene	Abbreviation	Subfamily designation	Tilapia	Spotted gar	Human
hydroxysteroid 11-beta-dehydrogenase 1	*hsd11b1*	SDR26C	2	1	2
fatty acid synthase	*fasn*	SDR27X	1	1	1
17-beta-dehydrogenase 1	*hsd17b1*	SDR28C	1	1	1
retinol dehydrogenase 8	*rdh8*	SDR28C	4	4	1
peroxisomal trans-2-enoyl-CoA reductase	*pecr*	SDR29C	1	1	1
17-beta-dehydrogenase 8	*hsd17b8*	SDR30C	1	1	1
sterol-4-alpha-carboxylate 3-dehydrogenase	*nsdhl*	SDR31E	1	1	1
dehydrogenase/reductase SDR family member 7B	*dhrs7*	SDR32C	5	3	3
dihydropteridine reductase	*qdpra*	qdpra	1	1	1
3-ketodihydrosphingosine reductase	*kdsr*	SDR35C	1	1	1
15-hydroxyprostaglandin dehydrogenase	*hpgd1*	SDR36C	4	2	1
3-keto-steroid reductase	*hsd17b7*	SDR37C	2	1	1
sepiapterin reductase	*spra*	SDR38C	1	1	1
epimerase family protein SDR39U	*sdr39u1*	SDR39U	1	1	1
dehydrogenase/reductase SDR family member 12	*dhrs12a*	SDR40C	3	2	1
WW domain-containing oxidoreductase	*wwox*	SDR41C	1	1	1
short-chain dehydrogenase/reductase family 42E	*sdr42e1*	SDR42E	2	2	2
flavin reductase	*blvrb1*	SDR43U	2	1	1
oxidoreductase HTATIP2	*htatip2*	SDR44U	1	1	1
carbonyl reductase 4	*cbr4*	SDR45C	1	1	1
17-beta-hydroxysteroid dehydrogenase 14	*hsd17b14*	SDR47C	1	0	1
nmrA-like family domain-containing protein 1	*nmral1*	SDR48A	0	1	1
versicolorin reductase	*vcr*	SDR112C	1	1	0
synaptotagmin-12-like	*syt12*	SDR348C	1	0	0
adenylate kinase 7-1	*ak7*	U	2	1	1
saccharopine dehydrogenase	*sccpdh*	U	1	1	1
uncharacterized LOC100690032	*c-factor*	U	2	2	0

Table S28 Expression (RPKM) of *SDR* genes in tilapia XX and XY gonad based on transcriptome data

gene	Transcript ID	5dXX	5dXY	30dXX	30dXY	3mXX	3mXY	6mXX	6mXY
hsdl1	ENSONIT00000023086	0	1.3	6.7	8.4	33.5	29	33	25.5
hsdl2	ENSONIT00000015800	31	35.7	50.8	47.4	57	85.3	67	110
hsd3b1	ENSONIT00000019776	1.8	1.8	379.5	348	61.5	1018.4	64.7	475.9
hsd3b7	ENSONIT00000012384	0	0	44	35.9	412.9	20.7	387	25.4
hsd11b1a-1	ENSONIT00000006128	5.5	0.8	1.9	1.3	0	0.5	0	0
hsd11b1a-2	ENSONIT00000006130	0.4	0	16	12	2.4	24.8	1.3	25
hsd17b1	ENSONIT00000001507	0	0	6	2	4.4	0	43	0
hsd11b2	ENSONIT00000004100	32.9	19	9.6	10.9	3.3	112	15.8	174.5
hsd17b3	ENSONIT00000019079	0	0.9	0	0.4	0	0.6	3.5	1.5
hsd17b4	ENSONIT00000018264	21.8	19	16	16.6	22.6	13.5	17.8	10.8
hsd17b7-a	ENSONIT00000003253	0	0	0.7	0.4	0.4	0.7	0	0.8
hsd17b7-b	ENSONIT00000023426	0.5	0	4.7	3	26.3	44.7	21.5	55
hsd17b8	ENSONIT00000025257	6.5	3	9	8.3	4.8	3.8	10	5.4
hsd17b10	ENSONIT00000020897	31	31	60.9	31	35.3	59	36	63.7
hsd17b12b	ENSONIT00000005286	0	0.4	0	0	0	0.8	0	0.3
hsd17b12a-a	ENSONIT00000019643	9.8	7.8	32	12.4	14	0.8	34	0.8
hsd17b12a-b	ENSONIT00000007424	20.6	21.9	17.4	18	8	45.6	16.8	30.4
hsd17b14	ENSONIT00000012251	18.3	34.4	12.3	15.7	1.6	7	6.4	6
rdh1	ENSONIT00000011095	5.6	6.3	6.7	4.8	0	0.3	0	0
rdh5	ENSONIT00000008639	0	0	0	0	0	0	0	0
rdh8b-2	ENSONIT00000002060	9.4	14.4	12.5	9.3	0	6	0.4	6.7
rdh8c	ENSONIT00000000237	3.7	196.3	0	0	0	0	0	0
rdh8a	ENSONIT00000024233	0	0	0.4	1	0	0	0	0
rdh8b-1	ENSONIT00000002087	0.4	0.3	2.6	2.9	0	6	0	3.4
rdh10-a	ENSONIT00000017990	124.7	125.9	36.8	51.7	21.4	25.5	17.8	26.9
rdh10-b1	ENSONIT00000005231	0	0.3	0.5	0.6	0	0.3	0	0.3
rdh10-b2	ENSONIT00000023906	0	0	2.5	1.7	61.7	0.8	72.3	0.8
rdh11-a	ENSONIT00000025715	17.7	13.5	7	9	18.8	39	16.5	7.4
rdh11-b	ENSONIT00000018230	7.8	24.4	0.3	0.9	0	0	0	0
rdh12b	ENSONIT00000001896	0	0.7	0	0.3	0	3.9	0	3.4
rdh12a	ENSONIT00000008045	20.7	21.3	28	18.3	151	24.8	143.3	21
rdh12c-b	ENSONIT00000024729	3.3	4.3	4.6	8	1.9	4.8	2.5	4.6
rdh12c-a	ENSONIT00000018225	0	0	0	0	0	0	0	0
rdh13b	ENSONIT00000025211	2.5	2	8.3	6.7	43	11.4	41.3	10.7
rdh13a	ENSONIT00000025211	6.4	7.5	8.3	6.7	43	11.4	41.3	10.7
rdh14-b	ENSONIT00000007412	0	0	7.5	6.5	20.3	124.7	25.7	125.5
rdh14-a	ENSONIT00000019693	0	0.5	7.6	9.3	45.4	15	65.8	12.7
dhrs3	ENSONIT00000000813	5.6	0	33.8	64	93.8	12.8	114	7
dhrs7b	ENSONIT00000025368	0.4	0.6	18	18.3	64	24.4	56.9	27
dhrs7c-a	ENSONIT00000025369	4.7	4.5	2.3	1.4	6	2	7	2
dhrs7c-b	ENSONIT00000024430	9.7	11.9	16.5	13.8	218.3	26.4	165.6	22.7
dhrs7a-1	ENSONIT00000012167	6	7.5	0.4	0	0	0.3	0	0
dhrs7a-2	ENSONIT00000024659	2	2.5	0.5	0.8	0	0	0	0.9
rdh7	ENSONIT00000011101	4	2	30	18.5	93.8	28.8	122	14.8
dhrs9	ENSONIT00000014934	24.3	21	30.5	13	13	29.8	18.7	36.7
dhrs11-b	ENSONIT00000016105	23.6	20.6	7.7	7	32.5	21	23.4	25.9

gene	Transcript ID	5dXX	5dXY	30dXX	30dXY	3mXX	3mXY	6mXX	6mXY
dhrs11-b2	ENSONIT00000016588	0	0	0	0	0	0	0	0
dhrs11-a1	ENSONIT00000016602	1	3.5	0.3	0	0	0	0	0.3
dhrs11-a3	ENSONIT00000016590	151.6	171.9	35.6	20	0.9	7.9	2	6.7
dhrs11-a6	ENSONIT00000016594	57	43	19.6	7	19	5.5	31	2.4
dhrs11-a5	ENSONIT00000017149	6.7	35.9	25.3	10.3	2	32.5	0	8
dhrs11-a11	ENSONIT00000016603	0.9	2	2.5	1	2.3	1.4	2	0.9
dhrs11-a13	ENSONIT00000016598	28.9	27.5	40.8	11	4.7	1.6	6.4	0.9
dhrs11-a8	ENSONIT00000005051	6.8	5	8.9	3.7	3.6	1.8	9	2.6
dhrs11-a7	ENSONIT00000016595	53	24.7	12.6	11.9	0.5	3.8	0.9	1
dhrs11-a10	ENSONIT00000016601	0	0.6	0	0	0	0	0	0
dhrs11-a12	ENSONIT00000016596	22	23	13	6.8	3.6	2	11	1
dhrs12-2	ENSONIT00000021551	35.9	38.4	25.9	29.3	16	31.3	17.6	28
dhrs12-1	ENSONIT00000012634	0	4	0.9	0.4	5	1.6	9.9	1
dhrs12c	ENSONIT00000018460	0	0	1.8	1.3	1.3	6	0.4	5.3
dhrs13a	ENSONIT00000002183	2	1.8	10.8	6.5	28.6	2	29	0.8
dhrs13a.3	ENSONIT00000010477	13.4	16.8	17.5	31	12.3	23.9	12	30
dhrs13b	ENSONIT00000010476	0.3	0.3	6.7	6	0.3	4.3	0.6	3.3
dhrsx1	ENSONIT00000009275	8.7	8.9	5.7	2.6	2.4	3.5	1	3.5
dhrsx2	ENSONIT00000005343	1.7	1	2.5	2.3	2.9	33	1.8	29.5
dhrs1	ENSONIT00000019583	7	2.5	12	11.9	1	9.5	0.8	8.3
dhrs2	ENSONIT00000023086	0	1.3	6.7	8.4	33.5	29	33	25.5
sdr39u1	ENSONIT00000015800	31	35.7	50.8	47.4	57	85.3	67	110
sdr42e1	ENSONIT00000019776	1.8	1.8	379.5	348	61.5	1018.4	64.7	475.9
sdr42e2	ENSONIT00000012384	0	0	44	35.9	412.9	20.7	387	25.4
rdhe2	ENSONIT00000006128	5.5	0.8	1.9	1.3	0	0.5	0	0
hpgd-b	ENSONIT00000006130	0.4	0	16	12	2.4	24.8	1.3	25
hpgd-a2	ENSONIT00000001507	0	0	6	2	4.4	0	43	0
hpgd-a3	ENSONIT00000004100	32.9	19	9.6	10.9	3.3	112	15.8	174.5
hpgd-a1	ENSONIT00000019079	0	0.9	0	0.4	0	0.6	3.5	1.5
qdpra	ENSONIT00000018264	21.8	19	16	16.6	22.6	13.5	17.8	10.8
cbr1a-1	ENSONIT00000003253	0	0	0.7	0.4	0.4	0.7	0	0.8
cbr1a-2	ENSONIT00000023426	0.5	0	4.7	3	26.3	44.7	21.5	55
cbr1a-3	ENSONIT00000025257	6.5	3	9	8.3	4.8	3.8	10	5.4
cbr1a-4	ENSONIT00000020897	31	31	60.9	31	35.3	59	36	63.7
cbr4	ENSONIT00000005286	0	0.4	0	0	0	0.8	0	0.3
blvrb	ENSONIT00000019643	9.8	7.8	32	12.4	14	0.8	34	0.8
gale	ENSONIT00000007424	20.6	21.9	17.4	18	8	45.6	16.8	30.4
kdsr	ENSONIT00000012251	18.3	34.4	12.3	15.7	1.6	7	6.4	6
ndufa9	ENSONIT00000011095	5.6	6.3	6.7	4.8	0	0.3	0	0
vcr	ENSONIT00000008639	0	0	0	0	0	0	0	0
far1	ENSONIT00000002060	9.4	14.4	12.5	9.3	0	6	0.4	6.7
tsta3	ENSONIT00000000237	3.7	196.3	0	0	0	0	0	0
syt12	ENSONIT00000024233	0	0	0.4	1	0	0	0	0
tdh	ENSONIT00000002087	0.4	0.3	2.6	2.9	0	6	0	3.4
tdh2	ENSONIT00000017990	124.7	125.9	36.8	51.7	21.4	25.5	17.8	26.9
tdh3	ENSONIT00000005231	0	0.3	0.5	0.6	0	0.3	0	0.3
spra	ENSONIT00000023906	0	0	2.5	1.7	61.7	0.8	72.3	0.8

gene	Transcript ID	5dXX	5dXY	30dXX	30dXY	3mXX	3mXY	6mXX	6mXY
uxs1	ENSONIT00000025715	17.7	13.5	7	9	18.8	39	16.5	7.4
bdh1	ENSONIT00000018230	7.8	24.4	0.3	0.9	0	0	0	0
bdh2	ENSONIT00000001896	0	0.7	0	0.3	0	3.9	0	3.4
wwox	ENSONIT00000008045	20.7	21.3	28	18.3	151	24.8	143.3	21
tgds	ENSONIT00000024729	3.3	4.3	4.6	8	1.9	4.8	2.5	4.6
blvrb1	ENSONIT00000018225	0	0	0	0	0	0	0	0
sccpdha	ENSONIT00000025211	2.5	2	8.3	6.7	43	11.4	41.3	10.7
mat2b	ENSONIT00000025211	6.4	7.5	8.3	6.7	43	11.4	41.3	10.7
nsdhl	ENSONIT00000007412	0	0	7.5	6.5	20.3	124.7	25.7	125.5
decr1	ENSONIT00000019693	0	0.5	7.6	9.3	45.4	15	65.8	12.7
decr2	ENSONIT00000000813	5.6	0	33.8	64	93.8	12.8	114	7
ak7-a	ENSONIT00000025368	0.4	0.6	18	18.3	64	24.4	56.9	27
gmds	ENSONIT00000025369	4.7	4.5	2.3	1.4	6	2	7	2
fasn	ENSONIT00000024430	9.7	11.9	16.5	13.8	218.3	26.4	165.6	22.7
htatip2	ENSONIT00000012167	6	7.5	0.4	0	0	0.3	0	0
ak7-b	ENSONIT00000024659	2	2.5	0.5	0.8	0	0	0	0.9
c-factor-a	ENSONIT00000011101	4	2	30	18.5	93.8	28.8	122	14.8
c-factor-b	ENSONIT00000014934	24.3	21	30.5	13	13	29.8	18.7	36.7

Table S29 Primers used for *ISH* and qPCR in this study.

Primer name	Primer sequence	Efficiency
rdh12b-qPCR-F	GGACCTCAACAGTGAGAAGC	1
rdh12b-qPCR-R	CGCCACAACTCAGTCTGAAC	1
sdr39u1-qPCR-F	CATGAACCCACTGCGATGGT	1
sdr39u1-qPCR-R	AGCAAGCTACACCTGACACC	1
far1-qPCR-F	GCCCTAAAACCCGCATCTCT	1
far1-qPCR-F	GGATGCTCTGGTCCGCTTTA	1
decr2-qPCR-F	GCACGGATGACTGTCTGACT	1
decr2-qPCR-R	TTCCTGCTGGCAATCACTG	1
hsd3b7-ISH-F	CGCGTAGATCCGACCTTGAA	
hsd3b7-ISH-R	AGGTGCATCCAGGCAACATT	
rdh12b –ISH-F	CGTTCAGATGACGCCACAAC	
rdh12b –ISH-R	CGTTCAGATGACGCCACAAC	
dhrs3–ISH-F	TACTTCCTGTGTGACGTGGC	
dhrs3 –ISH-R	TGCAACTTGTGGCATGAAGC	
dhrs7b –ISH-F	CTGTTGTCTTCGACCTGGCT	
dhrs7b –ISH-R	:AGAGTGTGCGGAGGTAGACT	
sdr39u1-ISH-F	GGTGACACTGATATCCCGCC	
sdr39u1-ISH-R	TGCGACTCCGTTGAACACT	
far1-ISH-F	TCCACAGCCTATGCCAACTG	
far1-ISH-R	TTAACATTGGGCCGACGGAA	
dcer2-ISH-F	ATGTTGGCACGGATGACTGT	
dcer2-ISH-R	AGATAGGACCTGGAGCCACA	
hpgd-b1 –ISH-F	ATGGTGGACCTGAACAAGGC	
hpgd-b1 –ISH-R	TCAGCGACGGCTGTAAAACT	
hsd11b2-ISH-F	CTGGGCTTTGAAGTGTTCGC	
hsd11b2-ISH-R	AGGTTGGTGGTGGTGTGTTT	
rdh10b-2–ISH-F	GCTGTGTCTGATCACGGGAG	
rdh10b-2–ISH-R	CTGCGAACATCCCTGTGTCT	
dhrs11-a6-ISH-F	GGGAAGAGTGGCTCTGGTGA	
dhrs11-a6-ISH-R	TGTGGAGCCGTGATGCAAAT	
ak7-a-ISH-F	CACCTGCTGGAGTGTGATGT	
ak7-a-ISH-R	CAATCGCTTCCTGTGGTGGT	
ak7-b-ISH-F	AAGAGGACCTTCCAGGTGGT	
ak7-b-ISH-R	CGCTGTAAACCACACTTGCC	
